# An economic model and evidence of the evolution of human intelligence in the Middle Pleistocene: Climate change and assortative mating

**DOI:** 10.1371/journal.pone.0287964

**Published:** 2023-08-02

**Authors:** Bruce C. Petersen

**Affiliations:** Department of Economics, Washington University, St. Louis, Missouri, United States of America; Sapienza University of Rome: Universita degli Studi di Roma La Sapienza, ITALY

## Abstract

A main objective of this paper is to provide the first model of how climate change, working through sexual selection, could have led to dramatic increases in hominin brain size, and presumably intelligence, in the Middle Pleistocene. The model is built using core elements from the field of family economics, including assortative mating and specialization and complementarities between mates. The main assumptions are that family public goods (e.g., conversation, shelter, fire) were particularly cognitively intensive to produce and became increasingly important for child survival during glacial phases. Intermediate climates (e.g., not the depths of severe glacial phases) create the largest gains from specialization, encouraging negative assortative mating. In contrast, severe glacial phases encourage positive assortative mating because of the rising importance of family public goods. One testable hypothesis is that absence of severe glacial phases should have led to stasis in brain size. Two other testable hypotheses are that severe glacial phases should have led to speciation events, as well as increases in brain size. The evidence shows that there was a million-year stasis in cranial size prior to the start of the severe glacial phases. This stasis is broken by a speciation event (*Homo heidelbergensis*), with the oldest fossil evidence dated near the close of the first severe glacial phase. In the next 300 kyr, there are two additional severe glacial phases, accompanied by considerable increases in cranial capacity. The last speciation event is *Homo sapiens*, with the earliest fossils dated near the end of the last of these two glacial phases.

## Introduction

This paper focuses on hominin evolution between 700 to 300 ka, a period of very sharp increase in both absolute and relative brain size [1,2], that followed nearly one million years of near stasis in brain size. During the 700 to 300 ka time period, the evidence also shows critical behavioral advancements, such as language capacity [3,4]. This paper makes a case that a few core economic principles, drawing particularly from the field of family economics, can help explain the evolution of complex cognition. With a few exceptions noted below, economic tools have not been used to help understand the evolution of hominins prior to *Homo sapiens*. This is unfortunate because, as emphasized by Robson and Kaplan [5], some economic tools are well suited for developing formal models of human evolution.

It has long been argued that climate change was an important driver of hominin evolution, with considerable attention given to glacial (cold) phases. For example, Calvin [6] emphasizes that ice ages likely greatly sped up gains in brain size and intelligence. Richerson and Boyd [7] state that the most obvious explanation for the rise of complex cognition is the deterioration of the Earth’s climate, particularly the glacial phases of the Middle Pleistocene. Stewart and Stringer [8] also emphasize the glacial phases of the Middle Pleistocene. Hughes and Gibbard [9] provide an overview of the glacial phases and Lisiecki and Raymo [10] provide commonly used dating, which is shown at the end of this paper. Marine Isotope Stage (MIS) 16 (676–621 kya) was the first severe glacial phase of the Pleistocene; subsequent severe glacial phases include MIS 12 (478–424 kya) and MIS 10 (374–337 kya). The most recent evidence [11] on temperatures in the Last Glacial Maximum shows global cooling of more than 6°C, on average. Such dramatic cooling would cause Southern Europe, the Levant, and North Africa, including Morocco, to experience very cold winter temperatures. In the Middle Pleistocene, these regions are key for understanding hominin evolution: i) the preponderance of hominin fossil evidence comes from Europe, especially Spain [12], ii) the Levant is the location of much of the best-documented evidence of the early control of fire [13,14] and iii) the earliest *H*. *sapiens* fossils are from Morocco, dated shortly after MIS 10 [15]. The present paper provides the first formal model showing how the glacial phases of the Middle Pleistocene could have been an important driver of complex cognition.

Sexual selection is a key component of the model. In *The Descent of Man*, *and Selection in Relation to Sex*, Darwin [16] placed great emphasis on sexual selection for hominin evolution. However, its role as an evolutionary force was then largely ignored for over a century [17,18]. Sexual selection and evolution is currently a major topic in biology, but there are no formal models of how it could impact the evolution of human intelligence. As documented by Miller [17, pp. 20], nearly all evolutionary theorists “have viewed natural selection as the exclusive director of human mental evolution,” with no attention to sexual selection. Miller [18] is an important and highly cited exception: he argues that intelligence evolved as a courtship tool, not for its impact on survival. The present paper takes a very different approach: complex cognition was a highly desired trait in the pair-bonding decision, as it contributed to child survival, a key argument in the family utility function.

This paper argues that sexual selection and parental cooperation, in conjunction with severe glacial phases, helped drive hominin intelligence in the Middle Pleistocene. Three assumptions underpin the model: i) there was a set of home-produced family public goods whose marginal productivity for child survival increased as the climate entered glacial phases, ii) this set of goods was particularly cognitively demanding to produce and iii) some pair-bonding began prior to the rise of *H*. *sapiens*. The set of family public goods includes fire, shelter, conversation, and child training [19]. Briefly, as described in the next section, the more severe the glacial phase, the more critical these goods became for preventing cold-related deaths caused by hypothermia [20,21]. Even in earth’s present non-glacial phase, cold-related excess human deaths are estimated to be several million annually [22], with a high fraction of all newborn deaths in many developing countries, including Africa, attributed to hypothermia [23].

In the model, gains from pair-bonding arise from family public goods, complementarities, and specialization by comparative advantage, all central building blocks in the literature on the economics of the family [19]. The family utility function includes private goods, children, and family public goods other than children. There is a child production function–with private goods (e.g., food) and family public goods as inputs–that determines the number of offspring that survive to adulthood. Finally, there are production functions for private and public goods in which males and females have two main traits, strength and intelligence, where the former is more important for private goods and the latter is more important for the production of family public goods.

The main results from the model can be easily summarized. Consider three types: 1) Type I is the most intelligent, but weakest, 2) Type II is intermediate and 3) Type III is the strongest, but least intelligent. Such a pattern is justified because of the enormous energy requirement of the brain [24], forcing tradeoffs between brain and muscle tissue; indeed, much of hominin evolution can be characterized as moving from robust body types to more gracile bodies with larger brains. During the brief interglacial periods, the value of family public goods is relatively low, and thus Type I are the least desired mates. For an intermediate climate (not severe glacial phases), public goods are now more valuable, and thus a more balanced mix of goods is optimal. Simulations of the model show that for reasonable differentials in trait values, there can be a substantial range of intermediate climate conditions where a Type I and Type III pairing (negative assortative mating, or NAM) generate the highest family utility. The intuition is that gains from specialization are greatest when a relatively balanced mix of goods is desired. A potential outcome is stasis in brain size. With sufficient deterioration in the climate, however, the optimal mix of production changes such that gains from specialization are overwhelmed by the benefits from high production of public goods, best achieved by a pairing of Type I (positive assortative mating, or PAM). Simulations suggest that the Type I pairing will not only have the highest fitness, but it may be the only pairing with child production high enough to survive a severe glacial phase.

Three main testable predictions are explored in the paper. The first is near stasis in hominin brain size in the milder (i.e., intermediate) climate conditions prior to the beginning of the severe glacial phases in the Middle Pleistocene. The other two predictions are that speciation events, together with increases in brain size, should appear in the fossil records near the trough of, or shortly after, severe glacial phases. As shown in the cranial capacity plot in the results section, there is an incredibly long period of near stasis in brain size prior to the severe glacial phases; this is consistent with the model as a period of milder climate swings is the most likely to generate a NAM outcome. This stasis is broken by a speciation event, *Homo heidelbergensis*, with the oldest fossil evidence dated near the end of MIS 16, the first severe glacial phase of the Pleistocene. Over the next 300 kyr, there are two additional severe glacial phases (MIS 12 and 10), accompanied by considerable increases in cranial capacity. The next speciation event is *H*. *sapiens*, with the earliest fossils dating to around 315 ka [15], shortly after the trough of MIS 10, consistent with the model.

This paper draws heavily from Becker [25–27] and Browning et al. [19]. Their treatments of the gains from “marriage” emphasizes complementarities and specialization by comparative advantage for joint production within the family. These gains–and their implications for assortative mating–were arguably of greater importance for humans in the Middle Pleistocene (compared to modern economies) given the far greater role of household production. Because models of human behavior are built around utility functions, a defense of the reproductive fitness advantages of utility maximization, building on the insights of Robson [28], is provided at the end of this paper.

Previous studies using basic economic tools to study hominin evolution before *H*. *sapiens* include Kaplan and Robson [29] and Robson and Kaplan [5,30]. They explore how the economics of hunter-gatherer societies could have led to the co-evolution of higher life expectancy and intelligence. They emphasize that, starting around 2 Ma, hunting large herbivores was cognitively demanding, raising the productivity of the brain. The focus of the present paper is much more recent, focusing on the Middle Pleistocene and *H*. *heidelbergensis*, typically argued to be the immediate predecessor of *H*. *sapiens*. Thus, some of the most cognitively demanding goods (e.g., language and fire) are arguably different. In addition, rather than motivated by the economics of hunting, the current paper focuses on family economics and evolutionary forces directly impacting both genders. A brief discussion of how the different models line up with fossil evidence on *Homo* evolution appears at the end of the paper.

Besides ecological theories and the models noted above, there are other explanations regarding the evolution of hominin intelligence. Wrangham [31,32] develops the “cooking hypothesis” which states that the invention of cooking, by greatly increasing the net energy extraction from food, had major impacts on hominin evolution, including brain size, particularly for early *Homo erectus* (e.g., 1.8 million years ago). Importantly, Wrangham [32, p. S308] explains why the cooking hypothesis likely does not explain the increase in brain size in the Middle Pleistocene.

Another example is the “social brain hypothesis” developed by Dunbar [33,34]. He argues that human intelligence arose not primarily to deal with environmental problems, but rather with the social challenges that arose from living in large and complex groups. Dunbar [34] emphasizes that monogamous pair-bonding is both highly socially complex and strongly associated with brain size in primates and certain other mammals. Given the importance of pair-bonding in the current paper, the model presented here complements the social brain hypothesis. Dunbar [33] presents evidence showing a correlation between average group size and the size of the neocortex for a large sample of primates. He does not, however, use the social brain hypothesis to explain the dramatic increase in hominin brain size in the Middle Pleistocene, together with the stasis proceeding this event.

The present study, by combining climate change with pair bonding and assortative mating, seeks to make progress understanding the large changes in brain size in the Middle Pleistocene. Without including climate change, it is challenging for theories of human evolution to explain the long period of stasis in brain size followed by the dramatic increase in brain size in the Middle Pleistocene. The evidence, while admittedly sharply curtailed by the limited number of known crania with capacity measures in the relevant time period, is consistent with the predictions of the model.

## Methods

### Three key assumptions

Additional details regarding the three main assumptions appear in the S1 File to this article. The first two assumptions involve family public goods, which includes language and control of fire, argued by Darwin [16] to be hominins’ greatest innovations. Fossil evidence (e.g., vocal tract and auditory capacity) of hominins in the Middle Pleistocene, such as *H*. *heidelbergensis*, indicates a language capacity roughly similar to modern humans [4]. Hlubik et al. [35] lists many studies providing evidence of the use of fire beginning in the early part of the Middle Pleistocene, with much of the best documented evidence of the early control of fire located in the Levant [13,14]. Shelter is another key family public good. Catt and Maslin [36, p. 1014] state that *H*. *heidelbergensis* would have built “tent-like” shelters with some of the earliest evidence dating to around 400 ka at Terra Amata in France [37].

The first key assumption is that the marginal productivity of family public goods for child survival increased as the temperature fell during glacial phases. A main justification for this assumption is that family public goods would surely become more critical for preventing cold-related deaths arising from hypothermia, which occurs when the body temperature falls below 35°C [38]. Hypothermia can kill very quickly (e.g., a matter of a few hours), unlike the lack of food, which typically requires weeks to kill a human. Zhao et al. [22] reports over 4 million global cold-related excess deaths annually, with Africa accounting for a large share of these deaths. A few decades ago, it was estimated that in developing countries, roughly 17 million newborns developed hypothermia annually with a very high incidence documented in parts of sub-Saharan Africa [39]. In a study conducted in Nepal, the risk of neonatal mortality increased 80% for each one degree below normal body temperature [40]. In Zambia, a majority of newborn deaths has been attributed to conditions (e.g., pneumonia) associated with hypothermia [23]. In the troughs of previous glacial phases, winter temperatures in much of Africa, the Levant, and Europe were much colder than in present conditions [11], arguably making hypothermia protection of children an even larger challenge.

Protecting children from hypothermia is achieved by a combination of fire and shelter, with a high-quality shelter trapping heat, protecting the fire, keeping children dry and blocking the wind. Hosfield [20], and especially Gilligan [21], emphasize the problem of hypothermia for Middle Pleistocene hominins in Europe, pointing to the importance of shelter and fire. The colder the climate, the greater the threat of hypothermia to children, and thus the greater the marginal productivity of fire and shelter for producing children that survive past childhood. The marginal productivity of language should also rise during a glacial phase. Language is often stated to be among the most critical tools as it facilitates cooperation, the coordination of activities, and the exchange of information [41,42]. A key example of exchange of information is child training (e.g., how to control fire and build effective shelters), and the survival benefits of this family public good logically increase as the climate deteriorates [7].

The second key assumption is that family public goods were more cognitively intensive to produce compared to private goods (e.g., food). Language and speech are often stated to be the most distinctive cognitive ability of humans [43], “amplifying the communicative abilities of *Homo sapiens* far beyond that of other Great Apes or any other species on this planet” [42, pp. 429]. Language and speech are also widely recognized as among the most cognitively complex functions [44,45], involving many regions of the brain, including both hemispheres [43,46]. Marslen-Wilson [47, pp. 424] describe the human language function as “a complex coalition of different neural systems of different evolutionary depths and origins.” Language has a strong genetic basis, involving many genes [48], and is clearly heritable [49].

Barkai et al. [50, p. S324] state that control of fire would have “necessitated elaborate knowledge-transmission mechanisms.” Twomey [51,52] argues that the control of fire was cognitively more complex than any other human behavior in the era when fire was first mastered, consistent with the control of fire being a “long and convoluted process” [53, pp. 1]. Twomey [51,52] also provides a framework for investigating the cognitive demands of using fire and concludes that hominins would have needed a protolanguage, well-developed working memory, and the ability to plan for uncertain future contingencies. Building an effective shelter is also cognitively challenging, as protection from hypothermia requires protection from wind and water.

Private goods production (e.g., food) is assumed to be a function of hominin strength, broadly defined. Multiple studies [54–56] have concluded that hominins have long engaged in “persistence hunting,” which means running the prey to exhaustion. Liebenberg [55] observed persistence hunting by contemporary hunter-gatherers and presents evidence that it can be a very efficient method of meat provision. Persistence hunting typically requires extreme endurance running, a trait that evolved by the time of early *H*. *erectus* [57]. In fact, Bramble and Lieberman [57] argue that critical aspects of the human body form arose because of the need for endurance running for either hunting or scavenging. Among primates, only humans are capable of endurance running and thanks to a diverse array of features, humans are much better endurance runners than most large herbivores [57]. In addition, scavenging, employed even by contemporary hunter-gatherers [58], as well as gathering, requires both strength and endurance.

This is not to say that hunting does not require cognitive ability. It should be emphasized, however, that humans are not the only primate that hunts in cooperative groups: beginning with Jane Goodall [59], there is overwhelming evidence that chimpanzees engage in organized hunts. At the same time, hominins are the only primates that developed the ability to do any of the following: 1) build sophisticated shelters, 2) control fire, and 3) master language. This suggests that producing family public goods is more cognitively demanding than procuring food from hunting. In addition, both females and males are extensively engaged in producing family public goods, placing selection pressures on both genders.

The third key assumption is that some Middle Pleistocene hominins were pair-bonding. Evidence on sexual dimorphism provides some insights, as polygynous higher primates display substantial sexual dimorphism while monogamous higher primates do not [60]. Many studies report substantial dimorphism in early *Homo* [61]. Importantly, there is considerable agreement that all hominins postdating *H*. *erectus* display skeletal size dimorphism similar to modern humans [61,62]. Based on this evidence, it is often concluded that Middle Pleistocene hominins (e.g., *H*. *heidelbergensis*) likely engaged in pair-bonding [62–64].

Chapais [65] states that the leading explanation for the switch to pair-bonding is the *parental cooperation* hypothesis, which is a family of models proposing that human pair-bonding arose to provide cooperative care of dependent children, including food provision and transfer of information. This description lines up well with evidence for contemporary hunter-gatherers, where fathers typically provide most of the protein [66,67], but mothers also provide a substantial proportion of the calories. Proponents of the importance of male parental contributions point to the long period of childhood dependency, requiring provisioning until the mid- to late-teens [62,64,66,68]. Catherine Key [69] computes the energetic costs of females to bear children, factoring in longer offspring dependency: she concludes that by ca. 500 ka, rising female energetic costs necessitated fathers’ participation in provisioning their own offspring.

Anthropologists have intensively studied mating systems of contemporary hunter-gatherer societies, which provides insights into the mating systems of earlier hominins. The evidence (see Crippen [70] for an overview) indicates that while polygyny is usually accepted, only a very small percentage of adult males have multiple (usually two) wives. Hunter-gatherer societies also have relatively high gender equality, which is important if females are to exercise choice in mate selection [71,72]. Marlowe [73] reports that fathers in contemporary hunter-gatherer societies provide higher levels of childcare than fathers in other societies, including industrial states.

### The basic model

The unit of analysis in the model is the individual (within the pair-bond), as evolutionary biologists have long argued that natural selection acts primarily at this level. Including the role of the group (e.g., members other than parents) would potentially enrich the analysis, but group selection theories of evolution are extremely controversial. For example, Nowak et al. [74] argue for the importance of group selection; a response, however, by 137 other evolutionary biologists [75] states that these arguments “are based upon a misunderstanding of evolutionary theory and a misrepresentation of the empirical literature.” Most behavioral biologists appear to be skeptical of group selection theories for human evolution.

The model below provides an illustration of how a few core principles from family economics, together with the assumptions discussed above, can explain how a climate switch to a glacial phase can drive the mating pattern from NAM to PAM. The model is very tractable, making it accessible to non-economists doing research in human evolution. In the model, *CHILD* is the (expected) number of children that survive to adulthood, *PUB* is family public goods other than children, while *PRIV* is private goods (e.g., food), which is allocated to both children (*PRIV*_*C*_) and to adults (*PRIV*_*A*_).

The production function for *CHILD* is Cobb-Douglas, commonly used in the literature for family production and utility functions [19,25,27]. This is the most algebraically tractable function that has two important properties that are sensible for the analysis: i) there is imperfect substitutability of inputs (i.e., diminishing marginal returns) and ii) a positive amount of both *PUB* and *PRIV* is required for positive *CHILD* production. Other functions with properties similar to Cobb-Douglas should deliver qualitatively similar predictions regarding the pattern of assortative mating. The function relating *CHILD* to public and private inputs is:

$$CHILD=K_{C}*{PUB}^{\Omega_{k}}*{\left({PRIV}_{C}\right)}^{\gamma_{k}}$$ (1)

Volk and Atkinson [76], based on a survey of twenty studies, report a child survival rate of only 51.2% for modern hunter-gatherers, motivating the critical problem facing Middle Pleistocene parents: how to transform the large number of births into children who survive to reproductive age. Eq (1) is thus best thought of as the production function at the most challenging stage of pair-bonding: multiple young offspring exist, and inputs must be provided so that some offspring survive to become *CHILD*. *K*_*C*_ is a climate “shock” which arguably declines in value during glacial phases due to higher incidence of hypothermia deaths, as described above. The subscript *k* indicates that the exponents on *PUB* and *PRIV* depend on climate conditions. As argued above, the marginal product of family public goods should rise considerably when the climate enters a glacial phase: shelter, fire, etc. become ever more important for preventing hypothermia deaths. The first key assumption is that the value of Ω_k_ rises (relative to γ_k_) as the climate worsens.

Eq (2) is a unitary family utility function, commonly used in family economics [77]. This function captures a young adult’s assessment of her/his future welfare when pair-bonded and children have arrived; this forward-looking view is standard in marital matching models, beginning with Becker [25]. The per-period flow of utility takes a standard Cobb-Douglas form:

$$U_{A}=K_{A}*[{PUB}^{\alpha_{k}}*{\left({PRIV}_{A}\right)}^{\beta_{k}}*{CHILD}^{\theta }]$$ (2)

*K*_*A*_ is the climate shock as it impacts adults, appearing only for symmetry with Eq (1) and having no impact on the main results. Children appear in the utility function, as it has long been emphasized that a main goal of marriage is to produce own children [19,25,27]. If one assumes that Ω_k_ in Eq (1) increases as climate deteriorates, a similar assumption is reasonable for α_k_ in Eq (2). However, this is not necessary, as choosing α and β to be independent of the climate does not change the main conclusions. For simplicity, θ is assumed to be independent of the climate (this has little impact on the results).

Substituting Eq (1) into Eq (2) leads to the following utility function:

$$U_{A}=K_{A}*[{PUB}^{\left(\alpha_{k}+\Omega_{k}\theta \right)}*{\left({PRIV}_{A}\right)}^{\beta_{k}}*{K_{C}}^{\theta }*{\left({PRIV}_{C}\right)}^{\gamma_{k}\theta }]$$ (3)

Family public goods impact adult utility through two channels: i) their own comfort (α_k_) and ii) the benefit arising from the impact on child survival (Ω_k_ θ). Parents make decisions regarding the mix of goods and also the allocation of private goods to parents versus children, a key tradeoff in a Malthusian world. So far, the only key assumption is that Ω_k_ varies with the climate as discussed above.

Now consider the production of private and public goods. Given the absence of markets, all goods are assumed to be home produced (arguably providing very strong incentives to engage in assortative mating). Hominins have two traits that matter for the production of goods: strength (S) and intelligence (T). As defended above, strength matters only for the production of private goods, while intelligence matters only for public goods in the basic model (this assumption is relaxed in the Supporting Information). Children would have acquired knowledge of production functions through daily observation of the production by parents and other adults. They would also observe that intelligence (e.g., protolanguage skills) is critical for producing goods like shelter and fire. That family public goods are more cognitively intensive to produce is the second key assumption.

Females have *H*^*f*^ hours and males *H*^*m*^ hours (and *H*^*f*^ *= H*^*m*^). Hours allocated to private and public goods are denoted by *h*^*f*^_*pri*v_ and *h*^*f*^_*pub*_ for females and *h*^*m*^_*pri*v_ and *h*^*m*^_*pub*_ for males. The budget constraints are given by *H*^*f*^ = *h*^*f*^_*pri*v_ + *h*^*f*^_*pub*_ and *H*^*m*^ = *h*^*m*^_*priv*_ + *h*^*m*^_*pub*_.

The production function for private goods for a mated pair is very basic and straightforward:

$$PRIV=h_{priv}^{m}*S_{m}+h_{priv}^{f}*S_{f}$$ (4)

Notice there are no complementarities (inputs are additively separable) in production. The justification is that contemporary hunter-gatherer couples typically do not interact when seeking food: males travel far afield (with a focus on meat) while females gather food closer to home.

In contrast, for public goods production, there are complementarities. Becker [25,27] emphasizes that complementarities are a key source of gains to marriage, and complementarities continue to be viewed as pivotal in the recent literature (see Browning et al. [19] for a review). The intuition for complementarities is that mated hominins would have interacted in the production of public goods like shelter, fire, conversation and the instruction of children. The complementarities involved in having a conversation are obvious. Building a shelter large enough to protect a family requires the hands (and brains) of multiple adults, and maintaining a fire is done by both mates. In what follows, complementarities arise only when mates work together, which is both realistic and tractable. While not necessary for the main results, complementarities add considerable insights because, as language skills improved, complementarities between mates would have become stronger. One insight (see below) is that stronger complementarities increase the benefits of PAM relative to NAM, causing PAM to be the equilibrium for longer periods over the climate cycle.

In the production function below, complementarities are captured by (*T*_*m*_ * *T*_*f*_)^ρ^, where ρ measures the strength of complementarities, a common approach in economics [25]. Note that gains from complementarities are greatest when both mates have high intelligence. For example, protolanguage skills are more valuable if both mates possess this skill, as planning, learning, and coordination is more effective.

The production functions for public goods depend on the pattern of assortative mating. Beginning with PAM, there are no gains from specialization by comparative advantage because mate traits are similar. Because complementarities in producing public goods is assumed to arise only when a couple works together, for any level of public goods, efficient production is for the female and male to allocate the same fraction of time to the production of public goods (to maximize complementarities). That is, *h*^*f*^_*pub*_ = *h*^*m*^_*pub*_ = *h*_*pub*_, where *h*_*pub*_ denotes the shared hours spent on public goods production. Thus, for PAM:

$$PUB=h_{pub}*{\left(T_{m}*T_{f}\right)}^{\rho }$$ (5)

For NAM, there is the potential for gains from specialization, a central concept in the family economics literature beginning with Becker [25]. Compared to modern economies where home production is typically modest [19], the potential for large gains from specialization (and NAM) were arguably much greater when nearly all goods were produced by individual households. To simplify the exposition, suppose the female is the most intelligent. (If the male is the most intelligent, the gender is reversed in the equations below.) When there is specialization, there are two cases:

$$PUB=h_{pub}^{f}*T_{f},\;when\;h_{pub}^{m}=0$$ (6.1)

$$PUB=h_{pub}^{m}*{\left(T_{m}*T_{f}\right)}^{\rho }+(H^{f}-h_{pub}^{m})*T_{f},\;when\;h_{pub}^{m}>0$$ (6.2)

In (6.1), only the female produces some public goods (the male specializes in private goods). In the second case (Eq 6.2), the female is fully specialized (*h*^*f*^_*pub*_ = *H*^*f*^) in public goods; if more are desired, then the male allocates some time to public goods and thus *h*^*m*^_*pub*_ > 0. Since complementarities arise only when both mates work together, complementarities are restricted to the minimum of (*h*^*f*^_*pub*_, *h*^*m*^_*pub*_), which equals *h*^*m*^_*pub*_. Once again, this amounts to the number of hours in public goods production shared by the couple. So, the first piece of Eq (6.2) captures the *PUB* production generated when complementarities are in play. All additional hours worked by the female, *H*^*f*^—*h*^*m*^_*pub*_, thus generate no complementarities, and this additional production shows up in the second piece of Eq (6.2). Eqs (6.1) and (6.2) are needed to delineate the two segments of the concave (to the origin) production possibility frontier when one of the partners is not completely specialized. Full specialization, the most interesting point, is where *PUB = H*^*f*^ * *T*_*f*_ and *PRIV = H*^*m*^ * *S*_*m*_. A more detailed discussion of the production possibility frontier for NAM is given below.

Trait variation is the raw material for evolution [78]. To push forward the analysis, consider the following three types: I = (S^L^, T^H^), II = (S^M^, T^M^) and III = (S^H^, T^L^), where L stands for low, M for medium and H for high, and this pattern exists for both males and females. The trait pattern above reflects a brain-muscle tradeoff: brain tissue has an energy requirement of about 16 times as much as that of muscle tissue by weight [24]. Indeed, there is evidence that hominins lost muscle to feed ever larger brains [79].

Consider now the optimal conditions for different mating patterns, beginning first with PAM. As noted above, with PAM, there are no possible gains from specialization. Thus, the optimal solution entails *h*^*f*^_*pub*_ = *h*^*m*^_*pub*_ = *h*_*pub*_, because for any given total time allocation to public goods, the gains from complementarities are maximized when both mates spend the same amount of time on public goods. For convenience, *h*_*priv*_ is thus defined as: *h*_*priv*_ = *h*^*f*^_*priv*_ = *h*^*m*^_*priv*_. The production function for public goods under PAM was given in Eq (5): $PUB=h_{pub}*{\left(T_{m}*T_{f}\right)}^{\rho }$. This means that the production function for private goods is: $PRIV=h_{priv}*(S_{m}+S_{f})$.

**Fig 1:**
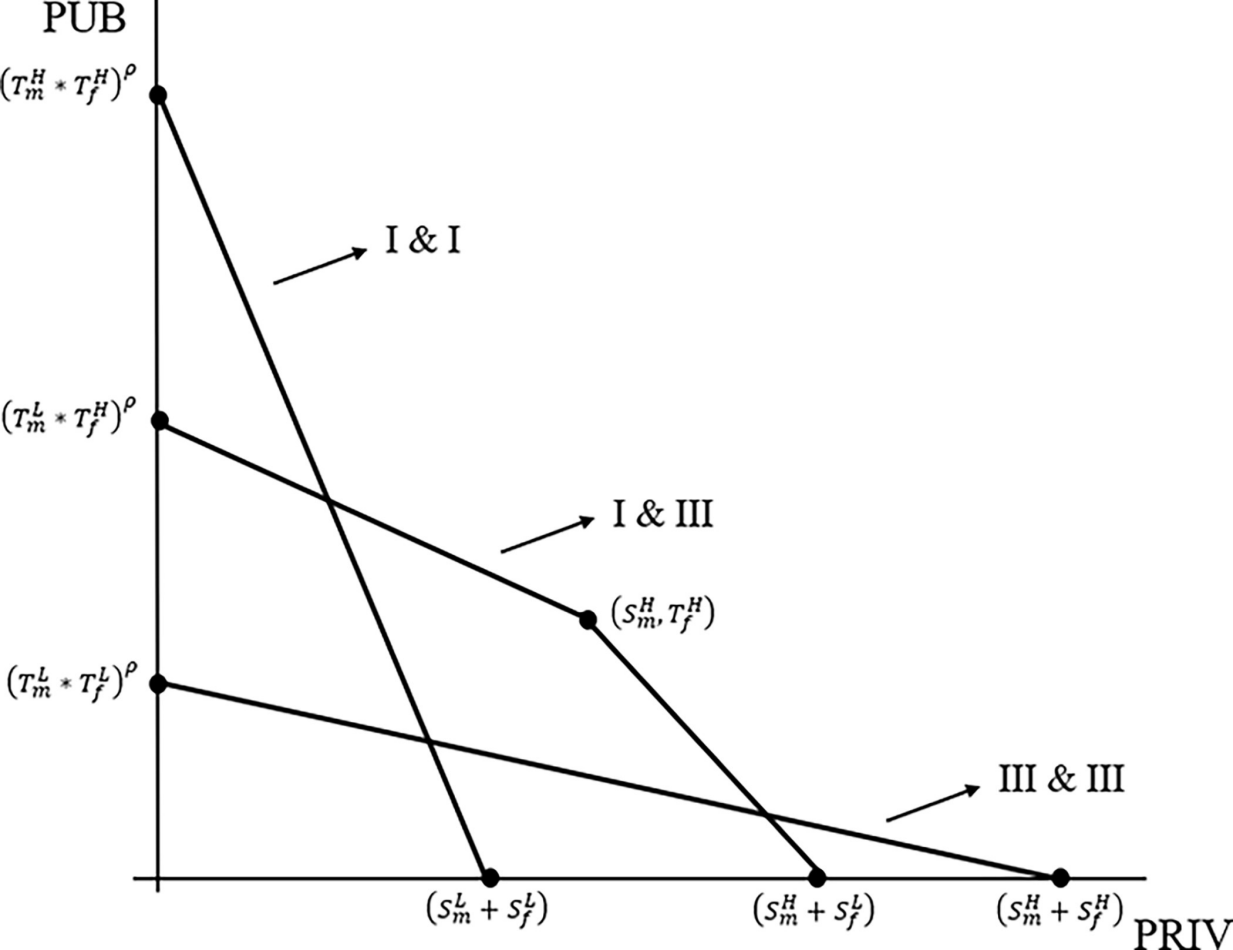
Production possibility frontiers for key pairings. The production possibility frontiers are for Types I & I, I & III, and III & III. In the I & III pairing, the female is Type I. Type I has traits (*S*^*L*^, *T*^*H*^), while Type III has traits (*S*^*H*^, *T*^*L*^). Hours are given by *H*^*m*^ = *H*^*f*^ = 1.

It is helpful to consider a graph of the family production possibility frontiers when PAM is occurring. The two key pairings are I and I and III and III, and their frontiers appear in Fig 1. The vertical axis is *PUB* while the horizontal axis is *PRIV*. To simplify the notation (with no loss of generality), assume *H*^*f*^ *= H*^*m*^ = 1. For the I and I pairing, the vertical intercept is ${\left(T_{m}^{H}*T_{f}^{H}\right)}^{\rho }$; for III and III, the vertical intercept is ${\left(T_{m}^{L}*T_{f}^{L}\right)}^{\rho }$. The horizontal intercepts are, respectively, $S_{m}^{L}+S_{f}^{L}$ and $S_{m}^{H}+S_{f}^{H}$. Increasing the strength of complementarities (ρ) rotates the production possibility frontiers upward (with the horizontal intercepts unchanged); this rotation is most pronounced for the Type I pairing, given that the vertical intercept is ${\left(T_{m}^{H}*T_{f}^{H}\right)}^{\rho }$.

Under PAM, hominins select the decision variables *h*_*pub*_ and *h*_*priv*_ to maximize utility (given in Eq 3) subject to the production functions above and the budget constraint *H* = *h*_*pub*_ + *h*_*priv*_, where the Lagrangian is given in the Appendix. This leads to the following optimality conditions:

$$PUB/{PRIV}_{A}=(\alpha_{k}+\Omega_{k}\theta ){\left(T_{m}*T_{f}\right)}^{\rho }/\left(\beta_{k}(S_{m}+S_{f})\right)$$ (7.1)

$$PUB/{PRIV}_{C}=(\alpha_{k}+\Omega_{k}\theta ){\left(T_{m}*T_{f}\right)}^{\rho }/\left(\gamma_{k}\theta (S_{m}+S_{f})\right)$$ (7.2)

$${PRIV}_{A}/{PRIV}_{C}=\beta_{k}/\gamma_{k}\theta$$ (7.3)

These relationships determine the optimal relative amounts of public and private goods to produce. The higher the benefit from public goods (α_k_ + Ω_k_θ) relative to private goods (either β_k_ or γ_k_θ), the greater the optimal level of family public goods relative to private goods. The greater the productivity of an hour of time devoted to public goods (*T*_*m*_ * *T*_*f*_)^ρ^ relative to the productivity for private goods (*S*_*m*_ *+ S*_*f*_), the greater the desired level of public goods. Thus, compared to other matches, a Type I pairing clearly results in higher public goods production for any given climate condition. Moreover, the optimal level of family public goods increases with the severity of the climate, as captured by the parameters Ω_k_ and α_k_. In addition to *h*_*priv*_ and *h*_*pub*_, the other decision variable is the allocation of private goods between adults and children, and this is determined by Eq 7.3.

Now consider the conditions when NAM is occurring and there is specialization by Types I and III. To simplify the discussion, assume the female is Type I (intelligent) and the male is Type III (strong). The concave (to the origin) production possibility frontier for the I and III pairing appears in Fig 1. With *H*^*f*^ *= H*^*m*^ = 1, the interior corner is given by $PRIV=S_{m}^{H}$ and $PUB=T_{f}^{H}$, while the horizontal intercept is $S_{m}^{H}+S_{f}^{L}$ and the vertical intercept is ${\left(T_{m}^{L}*T_{f}^{H}\right)}^{\rho }$. The male and female labeling is reversed if the types are reversed. For NAM, an important case is complete specialization by comparative advantage, the corner point in Fig 1. There are two other cases: i) the female producing some of both goods (along the lower line segment of the frontier and ii) the male producing some of both goods (along the upper line segment). The optimality conditions for these two cases are straightforward and are provided in the Appendix.

It is also noteworthy that complementarities for *PUB* production can be entirely removed from the analysis, instead utilizing a production function for *PUB* similar to the production function for *PRIV* (see Eq 4). If this is done, Fig 1 continues to have all the main features, including the concave frontier for the I and III pairing. PUB production would then be determined similar to PRIV production (see Eq 4); for example, for the I and I pairing, the vertical intercept is $T_{m}^{H}+T_{f}^{H}$.

What about pair-bonding of I and II or of II and III? In the simulations in the S2 File, these pairings are typically dominated by the other pairings. Compared to a pairing of I and III, there is less scope for specialization because of smaller differences in trait values. Unless complementarities are very weak, if either I and II or II and III pair bonds, the optimal production pattern is to set *h*^*f*^_*pub*_ = *h*^*m*^_*pub*_, which maximizes complementarities for any given time allocation to public goods. Optimal production then has the same setup as the PAM pairing described above.

Eqs (7.1) and (7.2), along with Fig 1, illustrate how climate conditions could impact assortative mating and reproductive fitness. As noted above, the higher the benefit from *PUB*, given by (α_k_ + Ω_k_θ), relative to private goods (either β_k_ or γ_k_θ), the greater the desired level of public goods relative to private goods. Note again that the only parameter that needs to vary with climate is Ω_k_. For brief interglacial periods, Ω_k_ is relatively low, and thus Type I is the least desired mating partner, given their comparative advantage in producing public goods. For an intermediate climate (colder, but not the depths of a glacial phase), public goods are now more valuable (higher Ω_k_), and thus a more balanced mix of public and private goods is optimal. It is thus plausible that there is a substantial range of intermediate climate conditions where a Type I and Type III pairing dominates the other two pairings, as depicted in Fig 1. The intuition is that gains from specialization are greatest when each type gets to spend all (or nearly all) of their time producing the good for which they have a comparative advantage. With sufficient deterioration in the climate, however, large values of Ω_k_ will eventually lead to such high marginal utility from *PUB* that gains from specialization are overwhelmed by the benefits from *PUB* production. That is, as suggested by Fig 1, at some point, Type I are best off pairing with each other, and thus pair bonding switches to PAM. Where this point occurs depends on the exact assumptions regarding the utility function, but it seems intuitive that with a sufficiently severe climate, and therefore large benefits from family public goods, the Type I pairing should eventual yield the highest family utility.

Many numerical examples are given in the S2 File and the results are intuitive. Pair-bonds are assumed to arise via the Gale-Shapley [80] Algorithm, commonly used for finding solutions to the stable matching problem, including pair-bonding [19]. The simulations begin with a warm climate and then let the climate deteriorate as follows: in the *CHILD* production function, lower *K*_*C*_ (the climate shock) and raise Ω_k_ (the exponent for *PUB*). The simulations in the S2 File show that for reasonable differences in trait values, there can be a substantial range of intermediate climate conditions where a Type I and Type III pairing generates the largest possible gain from pair-bonding (as suggested by Fig 1). However, as the climate becomes sufficiently severe (e.g., trough of a glacial phase), it becomes optimal for Type I to pair bond with each other. In the simulations, the switch to PAM occurs near the intersection of the production frontier of the Type I pairing with the upper line segment of the production frontier for the NAM pairing (in Fig 1). As the frontiers in Fig 1 suggest, once this switch to PAM occurs, the more severe the climate, the greater the relative advantage of the Type I pairing, including reproductive fitness. The simulations also show that the stronger are complementarities (captured by higher ρ), the quicker the switch from NAM to PAM, given that the gains from complementarities are greatest for the Type I pairing. This suggests that the development of language–which arguably raised the strength of complementarities–should have played a role in generating and maintaining Type I pairings.

Importantly, the simulations in the S2 File show that in periods of sufficiently severe climate, not only does the Type I pairing have the highest utility, but also the highest *CHILD* compared to all other pairings. This is expected, given that Type I has the comparative advantage in producing *PUB*, the input in the *CHILD* production function whose marginal productivity rises as the climate enters a glacial phase. This will be important for a prediction in the next section.

Also of importance, the simulations show that periods of severe climate are: i) particularly damaging to Type III *CHILD* production and ii) that only the Type I pairing may have *CHILD* production above replacement values, potentially leading to the elimination of all other types. Lower values of *K*_*C*_ in the *CHILD* production function during severe climate phases (e.g., more cold-related infant deaths) means that, other things equal, all pairings have lower *CHILD*. Lower *K*_*C*_, combined with a comparative disadvantage in *PUB*, is what can drive Type III (and possibly Type II) to extinction during a severe climate phase. In contrast, it is important to note that during mild climate phases, higher *K*_*C*_ means a lower likelihood of any type going extinct, including Type I, creating an asymmetry between severe versus mild climate conditions.

To summarize, the model captures, in a compact and straightforward fashion, how parental cooperation, specialization and complementarities could have played a role in driving assortative mating and reproductive fitness over the glacial cycles of the Middle Pleistocene.

## Results

### Timing of climate change

**Fig 2:**
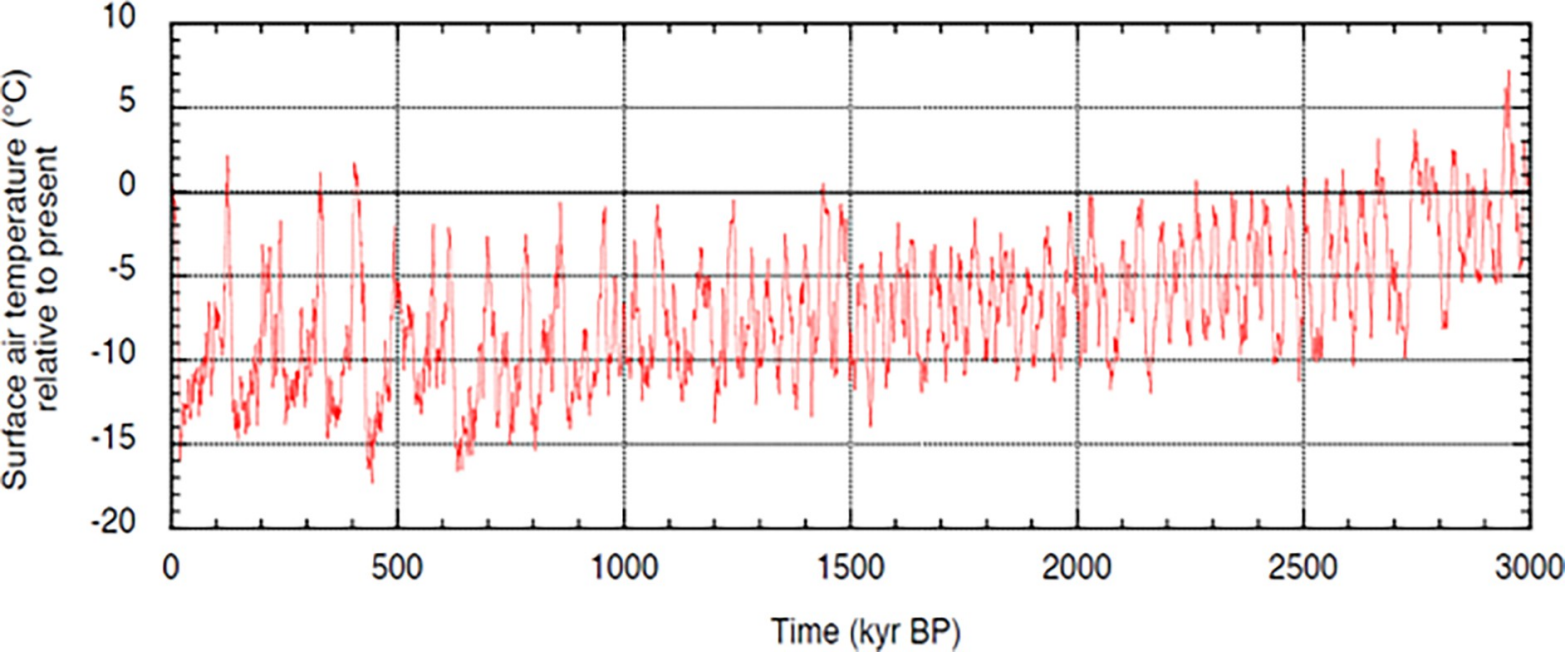
Surface air temperatures relative to present-day temperature. A plot of temperature in the Northern hemisphere (between 40° and 80° north) for the period 3 My to the present. The present temperature is normalized to 0°C. The plot was graciously provided by Richard Bintanja and is adapted from Fig 1B in Bintanja and Van de Wal [81]. See [82] for an in-depth discussion of their approach.

Fig 2, taken from Bintanja and Van de Wal [81], presents temperature estimates covering the last three million years. The vertical axis is annual average surface air temperatures (in °C) for the Northern Hemisphere continents between 40° and 80° north. These temperatures are relative to the present temperature, which is normalized to 0°C. One must look closely (tight to the left vertical axis) to see the 16°C jump in temperature in the last 12 kyr. Clearly, present-day temperatures are much warmer than those of nearly all periods in the last 1 Myr.

The temperature swings in Fig 2 are caused by three separate cycles (eccentricity, obliquity, and precession) that run simultaneously and impact the amount of sunlight reaching different parts of the Earth [83]. For much of the Pleistocene, climate cycles were roughly 41 kyr of length. Starting around 700 ka, there is a switch to a 100 kyr climate cycle, with much longer and colder glacial phases [9]. Maslin [83, p. 97] notes that with the switch to a 100 kyr climate cycle, the more severe glacial phases “have an increasing influence on the tropics, and in particular Africa.” This conclusion is supported by the recent evidence in [11], who report a very sharp drop in temperatures in much of Africa during the Last Glacial Maximum.

While the climate conditions of Africa are important in climate-driven theories of Middle Pleistocene human evolution, the climate conditions of Europe and the Levant are also key. There is growing support for the Assimilation Model of human evolution [84,85], which proposes admixture between migrating *H*. *sapiens* and earlier hominins living outside of Africa (see [86] for an overview). Furthermore, Papagianni and Morse [87, p. 51] note that “small groups were probably going in and out of Africa throughout much of the last two million years.” There is genetic evidence of both admixture and reverse migrations back into Africa and the Levant [88–90], with the Levant being a gateway in and out of Africa [88] and also an ice age refugium [91].

### Testable predictions

Consider first an adverse climate and the Type I pairing. Intelligence is a highly polygenic trait [92] and a large literature shows that intelligence is highly heritable (see [93] for a survey). Furthermore, studies show that the high end of intelligence is just as heritable [94]. A pairing of Type I should, on average, produce a Type I offspring. In a sufficiently severe climate, given the heritability of intelligence and the incentives for PAM, together with the highest fitness (i.e., highest *CHILD*) for the Type I pairing, the relative frequency of Type I should increase, leading to higher average intelligence in the population. This suggests **Prediction #1**: severe glacial phases should lead to an increase in intelligence, proxied by an increase in brain size.

Now consider the case of an intermediate climate resulting in NAM. A pairing of Types I and III should produce, on average, an intermediate type [95]. That is, there would be a tendency for assortative mating to drive the population to a more equal distribution of intelligence and strength. Thus, after a substantial period of NAM, most hominins would be about “average” (e.g., Type II). This suggests **Prediction #2**: periods of intermediate climate should lead to stasis in brain size.

NAM plays an important role in the model because it can explain the long stasis in brain size leading up to the Middle Pleistocene when climate swings were much milder. As can be seen in Fig 2, prior to around 800 kya, temperatures fluctuated in a much narrower band (intermediate climates). If this led to stasis in hominin evolution, what is the escape? In the model, what is required is a switch to a sufficiently severe glacial phase. This would provide incentives for PAM: individuals who were above average in intelligence (e.g., mutations or lucky genetic draws) would be best off seeking each other as mates.

Population bottlenecks are widely discussed in the evolutionary literature and are a prediction of the model. Hublin [96] states that the archeological record, as well as the paleo genetic evidence, suggests the glacial phases during the Middle Pleistocene led to multiple hominin population bottlenecks, which can lead to the complete elimination of less-fit genotypes [97]. A key result from the model is that the relative advantage in *CHILD* production of Type I gets progressively higher as the climate worsens, as Type I has the advantage in producing family public goods. For Type III, and to a lesser extent Type II, *CHILD* production is hit particularly hard by glacial phases. (See the pattern of below-replacement *CHILD* production for all pairings, except for the Type I pairing, in the adverse climate example in the S2 File). This implies that when there is a hominin population bottleneck during a glacial phase, it is plausible that only Type I survives, potentially leading to a speciation event. If so, when the climate reverses and becomes benign, although a Type III might now have a temporary advantage, it no longer exists. This suggests **Prediction #3**: hominin speciation events should occur around the time of troughs of severe glacial phases.

### Evidence: Speciation events and brain expansion

The predictions from the model are not dependent on a precise hominin classification in the Middle Pleistocene (which is both challenging and controversial), and thus the classification discussion below is brief. Many researchers argue that *H*. *heidelbergensis* is a distinct taxon that covers several of the Middle Pleistocene crania found in Africa and Europe [2,98,99]. *H*. *heidelbergensis* originated in either Africa or western Eurasia [1,100], with by far the most extensive fossil records occurring in Europe. The earliest fossil records are the Mauer 1 mandible from Germany, dating to around 609 ka, with evidence for inclusion in *H*. *heidelbergensis* reported in [96]. While it is commonly argued that *H*. *heidelbergensis* was the direct predecessor to *H*. *sapiens* [83,98,101,102], there continues to be debate in the literature. Remarkable new evidence has expanded both the range and antiquity of early *H*. *sapiens*. The evidence (including two crania) pushes the timing back to around 315 ka [15], with artifacts indicating control of fire and more advanced tools. The discovery, located in Morocco, led the investigators to conclude that the evolutionary processes of *H*. *sapiens* occurred over a vast landscape, including the Mediterranean region (an area heavily impacted by glacial phases of the Middle Pleistocene).

Evidence on absolute brain size over time is reported below. It is difficult to measure relative brain size because fossil limbs informative for body mass are relatively rare and typically not associated with skulls [1]. Importantly, evidence indicates little or no increase in hominin stature since the evolution of early *H*. *erectus* [103], suggesting that absolute brain size is an appropriate measure. While numerous cranial plots exist in the literature, they typically do not include the most recent discoveries; furthermore, previous plots do not show the timing of glacial phases. To construct a plot of cranial capacity, I begin with the database compiled in De Miguel and Henneberg [104]. Recent studies [e.g., 105,106] reporting cranial plots typically begin with the same database and then augment the data with the most recent discoveries. As commonly done, I report the average cranial size when there are multiple size measures and exclude juveniles. In addition, as done in other studies, I searched for all crania new to the literature (with data on size and age) and added 13 to the sample, as listed below. This includes five additional adult crania from Sima de los Huesos (in Spain), as well as the two *H*. *sapiens* crania from Morocco. Finally, I checked all crania for any modern re-dating. A list of the full set of crania is provided in the S8 File.

**Fig 3:**
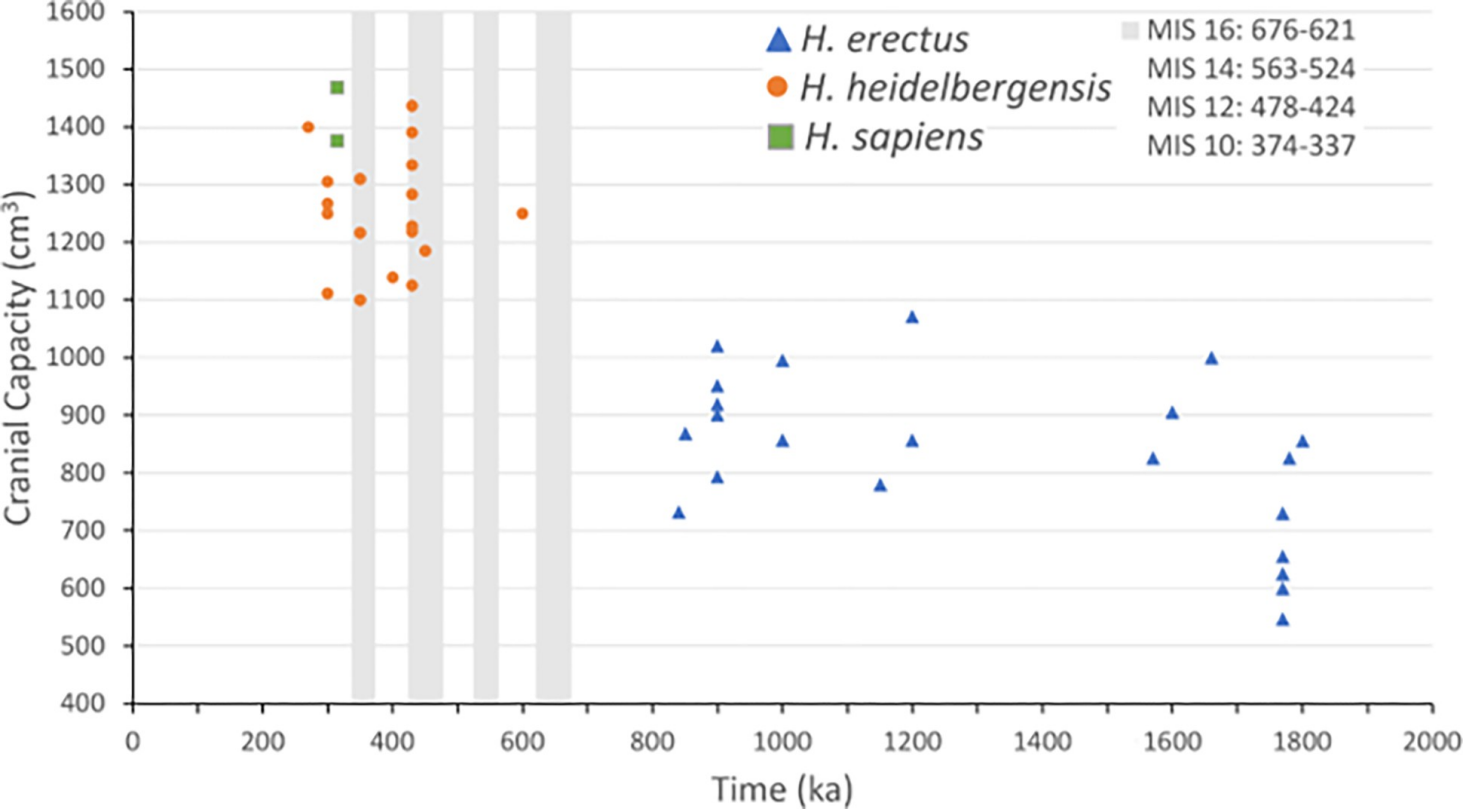
Cranial capacity plot for the time period 2 Ma to 270 ka. The demarcation of species and glacial phases are labeled in Fig 3. For observations taken from De Miguel and Henneberg [104]: i) the average cranial size is reported when there are multiple measures, ii) juveniles are excluded, and iii) two poorly dated crania are excluded (Ternifine and Reilingn), which were also excluded by [104]. There are thirteen additional crania: i) five additional from Sima de los Huesos [12], ii) the two *H*. *sapiens* crania from Morocco [15], iii) the five *H*. *erectus* crania from Dmanisi [107] and iv) the Daka *H*. *erectus* cranium [108]. The dating for the Ceprano cranium is taken to be 450 ka, as reviewed in [109]. The glacial phase dating come from Lisiecki and Raymo [10] and Lisiecki’s webpage, Ages of MIS Boundaries.

In Fig 3, the beginning date is 2 Ma and the ending date is 270 ka (there is a lengthy gap in the cranial record after 270 ka). Earlier than 700 ka, all observations are for *H*. *erectus* specimens, marked as triangles. After 700 ka, there are some late *H*. *erectus* crania from outside Africa (China and Indonesia), but these are less relevant for the predictions, and they are not included in Fig 3. After 700 ka, the next set of crania appearing in Fig 3 were referred to as “archaic humans” in De Miguel and Henneberg [104]; to reduce confusion, the crania after 700 ka (excluding *H*. *sapiens*), are labeled as *H*. *heidelbergensis* and are indicated by circles. The two *H*. *sapiens* crania (at 315 ka) are marked by squares. Finally, for 700–300 ka, the glacial phases are indicated by the grey columns. The dating of these phases is given in Fig 3.

Starting around 1,600 ka, there is little or no increase in cranial capacity for *H*. *erectus* for almost 1 Myr, similar to existing cranial plots in the literature (e.g., [1,83,106]). Rightmire [1] concludes that for the period of stasis shown in Fig 3, the archaeological record suggests very little change in either the anatomy or behavior for *H*. *erectus*. Since this stasis occurs during a period before the start of the severe glacial phases (i.e., MIS 16), it is consistent with the second prediction discussed above.

The stasis in brain size is broken by a speciation event, which is typically classified as *H*. *heidelbergensis* [100]. The earliest fossils are the Mauer 1 mandible from Germany (≈ 609 ka), and the Bodo cranium from Ethiopia (≈ 600 ka), the first non-*erectus* cranium in Fig 3. These fossils are dated shortly after the end of MIS 16 (676–621 kya), which, based on multiple measures, was the first severe glacial phase of the Pleistocene [9]. The next speciation event is *H*. *sapiens*, with the earliest fossils being the crania from Morocco at 315 ka [15]. As apparent in Figs 2 and 3, the two *H*. *sapiens* crania are dated shortly after the end of the trough of MIS 10 (374–337 kya). These speciation events support the third prediction discussed above.

Now consider the measures of cranial capacity reported in Fig 3. The Bodo cranium has a capacity of around 1250 cm^3^, much larger than the largest *H*. *erectus* cranium (≈ 1070 cm^3^) and far larger than the sample average (≈ 883 cm^3^) for *H*. *erectus* in Fig 3 after 1 Ma. There are no observations near MIS 14, the shortest and mildest glacial phase in the last 700 ka, and thus of less interest. There are eight crania dated around 430 ka, at the end of MIS 12 (478–424 kya), which, by multiple measures, was the most severe glacial phase of the Pleistocene [9]. While the average cranial size is 1275 cm^3^ (not substantially larger than the earlier Bodo specimen) three crania are distinctly larger. The two *H*. *sapiens* crania are dated shortly after the end of glacial phase MIS 10. Their average size is 1421 cm^3^, similar to modern humans. Overall, the evidence–while sharply limited by the number of crania in the fossil record–appears to line up reasonably well with the three predictions listed above.

## Discussion and conclusions

Consider again Robson and Kaplan’s [5,30] formal models of the economics of hunting and hominin intelligence, noted in the introduction. Their models can readily explain the large jump in brain size for early *H*. *erectus*, which appears in Fig 3. A model focusing on hunting, however, struggles to explain the following events: 1) the long stasis in *H*. *erectus* brain size and 2) the resumption of brain expansion in Africa and Europe near the start of the first severe glacial phase (MIS 16) of the Pleistocene. As noted in the introduction, without some consideration of climate change, it is challenging for any theory of human evolution to explain this pattern of hominin brain evolution.

Evolutionary biologists (see [110] for a review) have historically assumed that human behavior is hardwired by natural selection to maximize reproductive fitness. Economists obviously take an approach that is different from evolutionary biologists. In particular, Robson [28] presents a model showing that, compared to utility maximization, genetic hardwiring may be inferior because it potentially performs poorly when confronted with environmental fluctuations. In the chaotic climate of the last 700 kyr, genetically programmed rules arguably would likely perform poorly; in particular, upon entering a severe glacial phase, programmed rules could initially sharply under-produce family public goods, harming reproductive fitness. A numerical example of the reproductive fitness advantages of utility maximization appears in the S7 File.

Besides providing an economic model of the evolution of human intelligence in the Middle Pleistocene, this paper provides insights into three other topics in human evolution. First, the paper provides support for the long-standing claim that the rise of pair-bonding played a critical role in hominin evolution [65,111]. The paper also emphasizes an underappreciated explanation for the rise of pair-bonding: it has strong claims to being the most efficient mating system, as emphasized by Becker [25]. The literature on the economics of the family emphasizes efficiency gains from pair-bonding arising from specialization, complementarities, and assortative mating. These concepts play a central role in this paper’s model and arguably should be given more attention in theories of why and when hominin pair-bonding first became prevalent. An efficient mating system surely became ever more important with the lengthening of offspring dependency and the start of the severe glacial phases.

Second, many scientists have argued that the enormous advantages of both language [112,113] and fire [114,115] would have placed strong selective pressures on these behaviors. The present paper provides a model of how a selection process, operating through assortative mating, may have accelerated the acquisition of language and the control of fire.

Finally, this paper suggests that the economics of the family, particularly the focus on assortative mating as noted below, can be useful for future research on the evolution of sexual dimorphism in *Homo*. Grabowski et al. [116], employing the most comprehensive set of individual fossil hominin body mass predictions, concludes that early *H*. *erectus* exhibited high levels of sexual dimorphism. For *H*. *heidelbergensis*, two studies [117,118] of cranial and post-cranial variables (Atapuerca-SH sample) suggest a degree of sexual dimorphism similar to modern humans; however, another study [119] of mandibles of the Atapuerca-SH sample indicates levels of dimorphism higher than that for modern humans.

A prediction of this paper’s model is that the decline in body size dimorphism in *Homo* may have continued well into the time period of *H*. *Heidelbergensis*. The model indicates that periods of severe climate change, beginning with MIS 16, would have led to positive assortative mating. This means that mates are less specialized, in part because complementarities arise only when mates work together. With less specialization, females would likely engage in a higher degree of food provisioning which, if similar to patterns in modern hunter-gatherers, would involve females gathering large quantities of relatively low-calorie foods. In severe climate periods, the greater requirements for public goods, such as shelter, would also place greater transportation burdens on females if patterns of load transportation were similar to modern hunter-gatherers [120]. A recent study by Prado-Novoa et al. [120, pp. 7] finds no difference in the economy of load transport among sexes; rather, their results support a conclusion “that the distinctions between the economy of load transportation among sexes depends only on their differences in body mass.” This important finding, together with the results in the present study on assortative mating, suggests evolutionary pressures in the direction of larger female body mass (and thus a decline in sexual dimorphism) during glacial periods of the Middle Pleistocene.

To conclude, climate change is a leading explanation for the evolution of hominin intelligence, yet there are no formal models of how this might work. In addition, sexual selection, while heavily emphasized by Darwin [16], has been given little attention in the literature on the evolution of human intelligence. This paper considers how climate change, together with assortative mating and parental cooperation, could have sped up the evolution of complex cognition in Middle Pleistocene humans. The paper applies core economic principles, rarely used to explain human evolution prior to *H*. *sapiens*. The model shows that negative assortative mating can arise during non-glacial phases because of gains from specialization. Sufficiently severe glacial phases, however, cause family public goods–and therefore intelligence–to become valuable enough to drive sexual selection from negative to positive assortative mating. Once this switch occurs, the incentives for positive assortative mating, and the reproductive advantage of the most intelligent pairings, become ever greater the more severe the glacial phase. Evidence on speciation and stasis and increases in brain size is consistent with key testable predictions of the model.

Regarding hominin evolution, Robson and Kaplan [5, pp. 396] conclude that “there is a vast, untouched expanse of territory awaiting exploration in the adjoining hinterland of economics, biology and anthropology” where simple optimization models employed by economists can yield important insights. Their observation is even more relevant seventeen years later, given a wealth of new evidence concerning the abilities of Middle Pleistocene humans together with the locations and timing of hominin evolution.

## Supporting information

S1 Appendix (DOCX)

S1 File Additional information regarding the three assumptions. (PDF)

S2 File The main numerical examples: Climate, assortative mating and *CHILD*. (PDF)

S3 File Sensitivity of the numerical simulations to: 1) the exponent for *CHILD* in the utility function and 2) more than three types. (PDF)

S4 File Mating patterns for alternative trait values. (PDF)

S5 File Only public goods’ exponents vary with climate shocks. (PDF)

S6 File Allowing Intelligence to matter for private goods production. (PDF)

S7 File Defense of the use of utility functions (reproductive fitness). (PDF)

S8 File Crania used in Fig 3. (PDF)

S9 File Additional references. (PDF)
