# Supplementary material for: An economic model and evidence of the evolution of human intelligence in the Middle Pleistocene: Climate change and assortative mating
Source: PLoS One. 2023 Aug 2;18(8):e0287964. doi: 10.1371/journal.pone.0287964 (PMC10395973; doi:10.1371/journal.pone.0287964)
Supplement: S1 Appendix — (DOCX) [file pone.0287964.s001.docx]

**Appendix:**

**Lagrangian under PAM**:

$\mathcal{L}=K_{A}*\left[ {PUB}^{\left( \alpha_{k}+\Omega_{k}\theta\right)}*{{PRIV}_{A}}^{\beta_{k}}*{K_{C}}^{\theta}*{{PRIV}_{C}}^{\gamma_{k}\theta} \right]+\lambda\left[ H-\frac{PUB}{\left( T_{m}*T_{f} \right)^{\rho}}-\frac{{PRIV}_{A}}{\left( S_{m}+S_{f} \right)}-\frac{{PRIV}_{c}}{\left( S_{m}+S_{f} \right)} \right]$ where *H* = *h_priv_* + *h_pub_*. Given the production functions for *PUB* and *PRIV*, *H* = *H^f^* =  *H^m^.*

**Optimal Conditions under Negative Assortative Mating**

For Types I and III, besides complete specialization, there are two partial specialization cases. To simplify notation, assume the female is Type I (intelligent) and the male Type III (strong); if the types are reversed, so is the gender notation in the conditions below. One case is Type I producing both goods and Type III producing only private goods (with production of public goods given by equation 6.1 in the paper). The conditions that determine the optimal mix are:

|  | ${PUB}/{{PRIV}_{A}}= \left( \alpha_{k}+\Omega_{k}\theta\right)\left( T_{f} \right)/\left( \beta_{k}*S_{f} \right)$ | (1.1) |
| --- | --- | --- |
|  | ${PUB}/{{PRIV}_{C}}= \left( \alpha_{k}+\Omega_{k}\theta\right)\left( T_{f} \right)/\left( \gamma_{k}*\theta*S_{f} \right)$ | (1.2) |

Compared to (7.1) and (7.2) in the paper, the main differences are the absence of complementarities and the optimal mix being determined by the traits of one mate (the female in this case).

The other case is when Type I produces only public goods and Type III produces both goods (with production of public goods given by equation 6.2 in the paper). When the male is Type III, the optimal conditions are:

|  | ${PUB}/{{PRIV}_{A}}= \left( \alpha_{k}+\Omega_{k}\theta\right){\left( T_{m}*T_{f} \right)^{\rho}}/\left( \beta_{k}*S_{m} \right)$ | (2.1) |
| --- | --- | --- |
|  | ${PUB}/{{PRIV}_{C}}= \left( \alpha_{k}+\Omega_{k}\theta\right){\left( T_{m}*T_{f} \right)^{\rho}}/\left( \gamma_{k}*\theta*S_{m} \right)$ | (2.2) |

Compared to equations (1.1) and (1.2) above, one key difference is that there are now complementarities because both mates produce some public goods. Compared to equations (7.1) and (7.2) in the paper, only the trait of the male appears in the denominators.
