# Supplementary material for: An economic model and evidence of the evolution of human intelligence in the Middle Pleistocene: Climate change and assortative mating
Source: PLoS One. 2023 Aug 2;18(8):e0287964. doi: 10.1371/journal.pone.0287964 (PMC10395973; doi:10.1371/journal.pone.0287964)
Supplement: S1 File — (PDF) [file pone.0287964.s002.pdf]

S1: Additional information regarding the three assumptions

Insights into the antiquity of human language are obtained through fossil evidence of the vocal tract and the auditory capacity of Middle Pleistocene hominins. Martinez et al. [4], for Sima de los Huesos hominins, show that the pattern of sound power transmission (outer and middle ear) captures the frequencies relevant in modern human speech; they also report that the vocal tract proportion was capable of producing the human quantal vowels. The absence of air sacs in the vocal tract of *H. heidelbergensis* is also consistent with complex vocal communication [121]. Finally, evidence in de Boer et al. [122] indicates that language ability emerged gradually, through the accumulation of intermediate mutations.

The control of fire is commonly stated to be critically important in the cultural evolution and survival of hominins [114, 123]. Several authors date the habitual use of fire to the start of the Middle Pleistocene or even somewhat earlier [123 - 125]. Hlubik et al. [35] provide a long list of studies with evidence of the use of fire beginning in the early part of the Middle Pleistocene. Much of the best documented early evidence of the control of fire is found in the Levant [13], including the earliest evidence of the control of temperature to make flint blades [125] and evidence for the cooking of fish at Gesher Benot Ya'aqov in Israel 780,000 years ago [14].

One key assumption is that some Middle Pleistocene hominins were pair-bonding. Two points, not noted in the main text, are relevant. First, it is commonly noted [e.g., 126] that humans are the only primate that lives in multimale/multifemale communities where there is long-term pair bonding and extensive male parental care, suggesting an important role for pair-bonding in hominin evolution. Second, once pair-bonding arose, it would likely become the dominant mating system. The reason is that, compared to other mating systems, pair-bonding should result in a higher proportion of males who are both fathers and who have knowledge of their paternity; this should incentivize a greater proportion of males to provision (own) children, supporting larger group size. This, in turn, should lead to groups that engage in pair-bonding growing in size relative to groups not engaged in pair-bonding.
