# Supplementary material for: An economic model and evidence of the evolution of human intelligence in the Middle Pleistocene: Climate change and assortative mating
Source: PLoS One. 2023 Aug 2;18(8):e0287964. doi: 10.1371/journal.pone.0287964 (PMC10395973; doi:10.1371/journal.pone.0287964)
Supplement: S2 File — (PDF) [file pone.0287964.s003.pdf]

S2: The main numerical examples: Climate, assortative mating and *CHILD*

Simple numerical examples are often used in the economic literature [19, 26] to illustrate assortative mating patterns. The examples provided below seek to show that the basic model can generate intuitively plausible assortative mating patterns for reasonable parameter values. The main thing occurring in these simulations is changing Ω_k relative to γ_k (exponents in the child production function) as the climate changes. Pair-bonds are assumed to arise via the Gale-Shapley [80] Algorithm, commonly used for finding a solution to the stable matching problem, including pair-bonding [e.g., 19]. Each male seeks a female that provides him the greatest utility. Females receiving multiple offers reject all dominated offers and keep her top offer on hold. Rejected males make offers to their next highest-ranked female and so on. In the examples below, it makes no difference if the female (instead of the male) makes the proposals.

The first step is to consider the production possibility frontiers, which pin down the feasible production combinations for each pairing combination. Let the number of hours available to males and females be normalized to one ($H^f = H^m = 1$), for a total of two hours available to any pair-bonded couple. The number of hours chosen has no impact on the equilibrium mating patterns reported below, but it does matter for *CHILD* production. Given the hours normalization, the trait values are chosen such that *CHILD* exceeds replacement for some types over a wide range of climates. More precisely, the trait values are set such that *CHILD* (for the case of a benign climate) equals approximately Marlowe's [67] finding for the number of surviving children for contemporary hunter-gatherers (3.11 children). In the initial example, the traits (S, T) of the three types are set as follows: I = (3, 6); II = (4.5, 4.5) and III = (6, 3).

The T values should not be thought of as a cardinal measure of overall intelligence; rather, T measures the impact of intelligence on the production of public goods. For example, a modest gain in language ability (e.g., a favorable mutation as noted in [122]) may make a large difference in the production of shelter, child training, etc., given the importance of communication. Differences in trait values between Types I and III are important, as this determines the potential gains from specialization by comparative advantage. The modelling of complementarities (captured by the multiplication of T values) puts constraints on the set of reasonable values for T. Clearly, too small a T results in no complementarities and too large a T

would overwhelm gains from specialization for all mating combinations. This issue goes away, without changing the main results, if there are no complementarities in public goods production.

The other variable value needed to compute the production possibility frontier is ρ, the strength of complementarities. In the examples that follow, three values are considered: $\rho = 0.8$ (low), $\rho = 0.9$ (moderate), and $\rho = 1.0$ (high). For the trait values selected, these three ρ values generate a large range of gains from complementarities. For the initial example, $\rho = 0.9$. At this ρ value, gains from complementarities are moderate in size.

Fig S1 contains three production possibility frontiers with private goods (*PRIV*) on the horizontal axis and public goods (*PUB*) on the vertical axis. The frontiers are for the key combinations: I and I, III and III and I and III. In this initial example, for all climates, the II and II combination is always dominated by one of the combinations listed above. For each frontier, the horizontal intercept is $PRIV = S_m + S_f$ while the vertical intercept is $PUB = (T_m * T_f)^{\rho}$ where $\rho = 0.9$. So, for the III and III pairing, the horizontal intercept is 12 and the vertical intercept is $(3 * 3)^{.9} = 7.23$. For the two PAM pairings, there is no specialization and thus the frontiers are straight lines. For the I and III pairing, the production frontier exhibits the typical concavity (to the origin) due to specialization. In the lower segment, there are no complementarities, as only Type I produces public goods, as captured by equation 6.1; in the upper segment, there are complementarities, as Type III also produces public goods, as captured by equation 6.2.

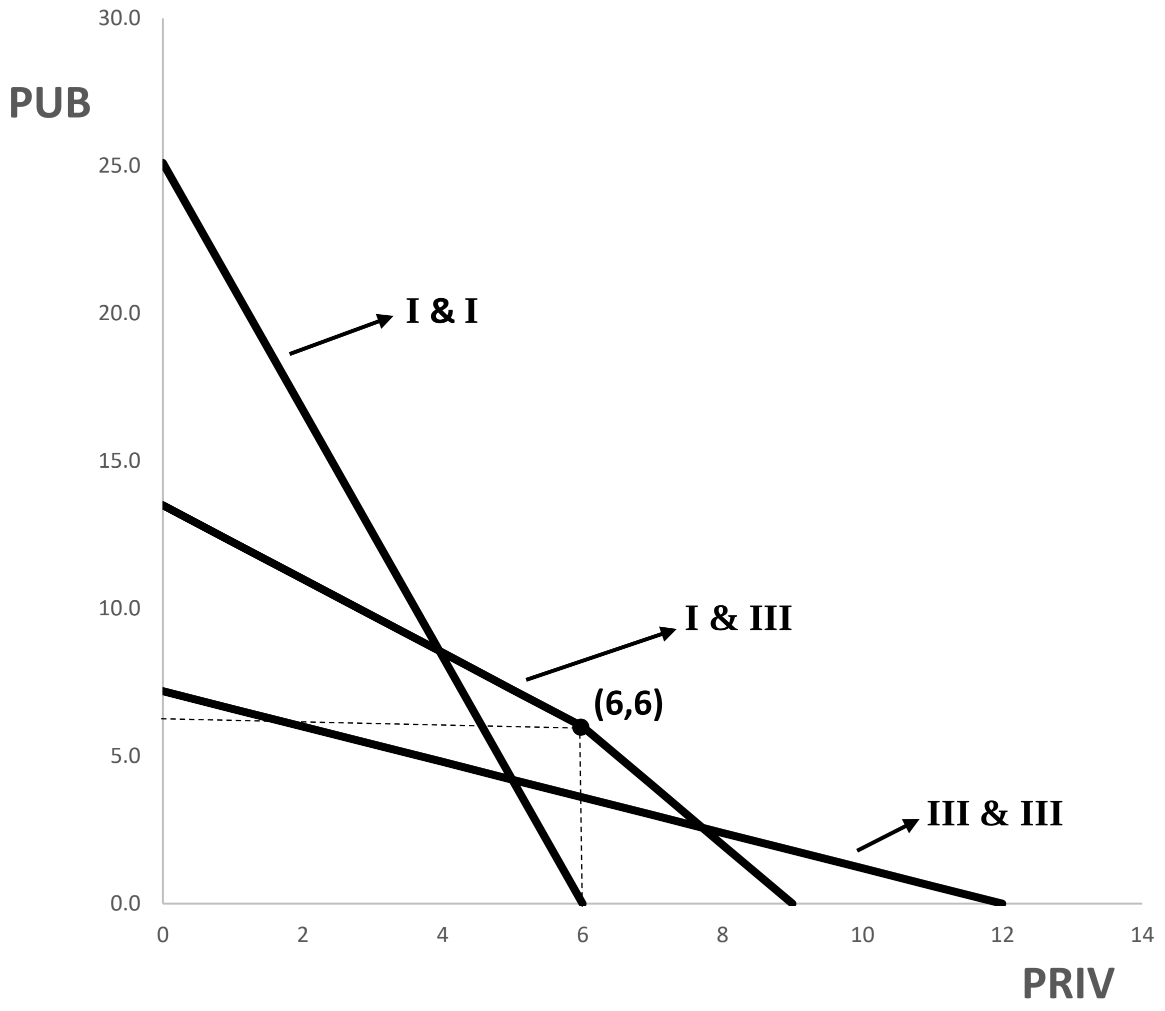

Fig S1. Production Possibility Frontiers for Key Pairings

PRIV and *PUB* are the production levels for private and public goods. The traits are (3,6) for Type I and (6,3) for Type III. Each type has hours given by $H^f = H^m = 1$. The equations for the production functions are given in the main text.

The production possibility frontiers in Fig S1 help clarify what pattern of assortative mating to expect. Starting with a benign climate (e.g., $\Omega_k \approx 0$ and $\alpha_k \approx 0$), public goods have low benefits. Thus, Type III seeks each other as mates, given their comparative advantage in producing private goods. As the climate deteriorates, public goods become more valuable, and thus a more balanced mix of public and private goods becomes optimal. This implies a range of climate values where it is optimal for Types I and III to pair bond. Finally, in a sufficiently

severe climate, because the benefits of public goods are high, optimal production is heavily weighted towards public goods, where a Type I pairing dominates.

Three sets of parameter values are given in Table S1, chosen to generate assortative mating outcomes (reported in Table S2A) that are well away from the boundaries. (Mating patterns for a continuum of climate values are reported in Table S3.) The nature of the climate is summarized by K_C and K_A, the climate shocks in the *CHILD* production and the adult utility functions, respectively. For simplicity, $K_C = K_A$ in all examples. Three climates are considered: (1) **benign**, where $K_C = 0.85$; (2) **intermediate**, where $K_C = 0.65$; and (3) **adverse** where $K_C = 0.45$. The adverse climate corresponds to the very depths of severe glacial phases (e.g., the protracted troughs of ice ages; see Figs 2 and 3 in the main paper). As noted in the paper, both K_C and K_A are irrelevant for the assortative mating outcomes.

Table S1: ***Parameter Values as a Function of Climate: An Example***

	K_C	K_A	Ω	γ	α	β	θ	ρ
**Benign Climate**	0.85	0.85	0.15	0.85	0.075	0.425	0.5	0.9
**Intermediate Climate**	0.65	0.65	0.35	0.65	0.175	0.325	0.5	0.9
**Adverse Climate**	0.45	0.45	0.55	0.45	0.275	0.225	0.5	0.9

As discussed above, in the *CHILD* production function, Ω_k, the exponent for public goods, should rise relative to γ_k, the exponent for private goods, as the climate enters a glacial phase. In Table S1, it is assumed that there are constant returns, or $\Omega_k + \gamma_k = 1$, which is a useful benchmark. This implies that γ_k declines as the climate deteriorates. An alternative approach is to fix γ_k and allow only Ω_k to vary with the climate. As shown later in the Supporting Information, this has no important impact on the simulations.

The third through sixth variables in Table S1 move lockstep with changes in the values for K_C and K_A. The exponent for public goods in the *CHILD* production function is set equal to

$\Omega_k = 1 - K_C$; this is for convenience, as all that is required is that Ω_k rises as the climate deteriorates. Assuming constant returns, this means that $\gamma_k = K_C$ (exponent for private goods in the *CHILD* production function). Next, the exponents for public goods (α) and private goods (β) in the utility function are set proportional to the respective exponents for public goods and private goods in the *CHILD* production function. For simplicity, a parent's utility from children is independent of climate: in the seventh column, the exponent for *CHILD* in the utility function is fixed at $\theta = 0.5$. (The pattern of assortative mating is not sensitive to substantial changes in θ, as shown later in the Supporting Information.) Given that $\theta = 0.5$, then $\alpha = \Omega_k/2$ and $\beta = \gamma_k/2$, consistent with the assumption that $\alpha + \beta + \theta = 1$. Thus, the impact of climate change, as it impacts adults and children working through public and private goods, is treated symmetrically. This is not important, as the main results arise even if α and β do not vary with the climate.

Tables S2A and S2B report values for utility and *CHILD*, respectively, generated by the model, using the values selected for the traits (see above) together with the parameter values in Table S1, for all nine mating combinations and each of the three climates. For the I and III pairing, partial specialization is optimal in the benign climate and full specialization in the other two climates. In all other pairings, the values appearing in the tables are generated by the production functions for the PAM pairings (e.g., equation 5 in the main text) with the optimal mix of goods given by equations (7.1) and (7.2) in the main text. Because males and females of the same type have the same trait values, the off-diagonal numbers in the lower-left corner have corresponding values in the upper-right corner.

Table S2A: ***Utility Outcomes by Mating Pattern and Climate***

Benign (K_C =.85; Ω =.15)				Intermediate (K_C =.65; Ω=.35)				Adverse (K_C =.45; Ω =.55)			
	Female				Female				Female		
**Male**	**I**	**II**	**III**	**Male**	**I**	**II**	**III**	**Male**	**I**	**II**	**III**
**I**	2.12	2.46	2.84	**I**	1.73	1.83	2.00	**I**	1.47	1.41	1.33
**II**	2.46	2.77	2.99	**II**	1.83	1.88	1.82	**II**	1.41	1.32	1.16
**III**	2.84	2.99	3.17	**III**	2.00	1.82	1.76	**III**	1.33	1.16	1.01

In Table S2A, consider first the benign climate, the least interesting case because interglacial periods are very brief. (See Fig 2 in the main text.) In this case, all three male types "propose" to Type III females, who select a Type III male. Once Type III males are unavailable, in the next round of matching, Type II females accept the proposals of the Type II males. Equilibrium pair-bonding is thus the downward sloping diagonal. This is PAM, with Type I the least-preferred partner.

In contrast, in the intermediate climate (e.g., cold, but not the trough of ice age), the Type I male is best off with the Type III female, who in turn is best off with him; likewise, the Type III male is best off with the Type I female and she is best off with him. Thus, pair-bonding is NAM, and the intuition for why this occurs in the intermediate climate is that the gains from specialization are greatest when a balanced set of goods are desired. What if there are fewer Type I than Type III? Then all Type I match with Type III and the un-matched Type III seek mates other than Type I. The key point is that Type I are matched with the less intelligent type. Second, are the gender patterns of specialization problematic when a Type III female is paired with a Type I male? It may be difficult for the female (with young infants) to specialize entirely in private goods, as hunting far afield is impractical; so, the Type I male may need to provide

some private goods. That said, Jarvenpa and Brumbach [127] emphasizes considerable interchangeability of gender roles among hunter-gatherers, including food procurement. In any event, it does not fundamentally change the NAM result for the intermediate climate in Table S2A: the Type I female is best off with the Type III male, and the Type II female is best off with the Type II male, leaving the Type III female with only the Type I male. The main conclusion is that the intermediate climate does not provide incentives for PAM.

Finally, for the adverse climate in Table S2A, each type's utility is highest when pairing with a Type I because Type I excels at producing public goods, leading to Type I pairings at the end of the first round of matching. In the second round, the Type II female accepts the Type II male. Thus, PAM is the equilibrium mating pattern. This outcome highlights the importance of female choice, as the strongest males achieve higher utility if they are able to pair with Type I females. The rise of pair-bonding arguably supported young females making mating choices, as fathers would now know their offspring, as emphasized by Chapais [63].

A key difference between the PAM outcomes in the benign and adverse climates is that in the latter, Type I is now the most preferred partner. Notice also that the utility for the Type I pairing is least impacted by climate. The intuition is that Type I individuals have a comparative advantage in producing family public goods, whose importance rises as the climate deteriorates, partially offsetting the negative climate shock to K_A.

In Table S2B, the pattern for *CHILD* production by type pairings and climate is exactly as expected. In the benign climate, the Type I pairing has the lowest *CHILD* while the Type III pairing has the largest. Critically, this is reversed in the adverse climate. Importantly, *CHILD* for the Type I pairing is the least impacted by adverse climate conditions given that Type I has a comparative advantage in producing family public goods. Thus, as the climate worsens, the relative advantage in *CHILD* production of the Type I pairing gets progressively larger, which is critical, as this is the most challenging period to produce *CHILD*. (See equation 1 in the main text.) This suggests that in a sufficiently adverse climate period, *CHILD* production by other types may fall below replacement rates (as in Table S2B), leading to their extinction, as emphasized in the next section.

Table S2B : ***CHILD Production by Mating Pattern and Climate***

Benign (K_C =.85; Ω =.15)				Intermediate (K_C =.65; Ω=.35)				Adverse (K_C =.45; Ω =.55)			
	**Female**				**Female**				**Female**		
**Male**	**I**	**II**	**III**	**Male**	**I**	**II**	**III**	**Male**	**I**	**II**	**III**
**I**	2.30	2.67	3.09	**I**	2.15	2.27	2.49	**I**	2.18	2.10	1.98
**II**	2.67	3.00	3.24	**II**	2.27	2.33	2.26	**II**	2.10	1.97	1.73
**III**	3.09	3.24	3.44	**III**	2.49	2.26	2.18	**III**	1.98	1.73	1.50

For the same trait values used in making the calculations in Tables S2A and S2B, now consider the pattern of assortative mating as a function of climate and different strengths of complementarities. Changing climate amounts to starting at $K_C = K_A = 1$ and moving towards zero and changing in lockstep the other parameter values in Table S1 (e.g., $\Omega_k = 1 - K_C$ and $\gamma_k = K_C$). Table S3 reports the climate range, measured by Ω_k, for the initial PAM region, followed by NAM, and then the final PAM region, for a substantial range of strength of complementarities. The focus is on the NAM region and the final PAM region.

Table S3: ***Mating Patterns Across Climate Conditions with Different Complementarities (ρ)***

ρ	**Initial PAM**	**NAM**	**I & II**	**Final PAM**
0.80	$\Omega < 0.20$	$0.20 < \Omega < 0.62$	--	$\Omega > 0.62$
0.90	$\Omega < 0.24$	$0.24 < \Omega < 0.48$	--	$\Omega > 0.48$
1.00	$\Omega < 0.29$	$0.29 < \Omega < 0.38$	$0.38 < \Omega < 0.44$	$\Omega > 0.44$

The results for $\rho = 0.90$, the case explored in Tables 2A and 2B, are reported in the middle row of Table S3. NAM occurs between $0.24 < \Omega < 0.48$ and the second PAM region begins when $\Omega > 0.48$. The size of the NAM range is roughly as expected, given the location of the three production frontiers in Fig. S1. While not reported in Table S3, where mating switches to the final PAM region ($\Omega \approx 0.48$), the Type I pairing produces 2.17 *CHILD*, the Type II pairing produces 2.07 *CHILD* and the Type III pairing produces 1.68 *CHILD*. This below replacement outcome for Type III is important for considering the evolution of intelligence across climate cycles.

The first row of Table S3 considers $\rho = 0.80$ (weak complementarities). Given that $PUB = (T_m * T_f)^{\rho}$, a reduction in ρ rotates downwards the entire frontier for each of the two PAM pairings in Fig S1 above. For the I and III pairing, the lower portion of the production frontier is unchanged; only the upper segment is impacted since this is where both mates produce some public goods and thus complementarities come into play. Thus, reducing ρ negatively impacts the two PAM pairings more than the NAM pairing, and thus the size of the NAM region must increase, as shown in Table S3. The NAM region becomes even larger if there are no complementarities for public goods production: for $PUB = h^{m}_{pub} * T_m + h^{f}_{pub} * T_f$, the NAM region becomes $0.21 < \Omega < 0.79$.

The last row of Table S3 is for $\rho = 1.00$, which generates large gains from complementarities, causing the NAM region to shrink ($0.29 < \Omega < 0.38$). The I and II pairing dominates all other pairings between $0.38 < \Omega < 0.44$. (This pairing did not come into play in Fig

S1 when $\rho = 0.90$.) The I and II pairing is a transition phase before the final PAM region, which begins at $\Omega > 0.44$.

In the final PAM region of Table S3, the Type I pairing has the greatest *CHILD*. After entering the final PAM region, the larger is Ω, the greater is the advantage in *CHILD* production enjoyed by the Type I pairing. Thus, the incentives for PAM get progressively stronger as the climate worsens. Finally, increasing the strength of complementarities has the greatest impact on the Type I pairing, causing the NAM region to shrink and the final PAM region to expand. This is important because there is fossil evidence [4] that language ability was still improving during the Middle Pleistocene, arguably increasing the strength of complementarities in the production of public goods, increasing the fitness advantages of the Type I pairing during glacial phases.

One final point should be made about complementarities in public goods production. If they become very strong, eventually the gains from complementarities overwhelm the gains from specialization. In this case, optimal production for I and III is determined by (4) and (5) in the paper and the mix of public and private goods by (7.1) and (7.2).
