# Supplementary material for: An economic model and evidence of the evolution of human intelligence in the Middle Pleistocene: Climate change and assortative mating
Source: PLoS One. 2023 Aug 2;18(8):e0287964. doi: 10.1371/journal.pone.0287964 (PMC10395973; doi:10.1371/journal.pone.0287964)
Supplement: S3 File — (PDF) [file pone.0287964.s004.pdf]

S3: Sensitivity of the numerical simulations to: 1) the exponent for *CHILD* in the utility function and 2) more than three types

Consider first the sensitivity of the simulations to changes in the importance of *CHILD* in the utility function, the one variable in Table S1 that was kept constant (at $\theta = 0.5$) as the climate changed. The ranges of NAM and PAM exhibit little sensitivity to substantial changes in the θ value. For example, set $\rho = 0.9$ (the baseline value for complementarities) and maintain $\alpha + \beta + \theta = 1.0$ for the utility function. If $\theta = 0.4$, the NAM range is $0.25 < \Omega < 0.48$. Alternatively, if $\theta = 0.6$, the NAM range $0.24 < \Omega < 0.49$. These ranges are very similar to the NAM range for $\rho = 0.9$ reported in Table S3 above.

Second, what if there are more than three types, with additional types taking on values intermediate to the three types used in the paper? The size of the NAM region, and the start of the final PAM region, barely change. For example, set $\rho = 0.9$ and retain the three initial types. Now add two intermediate types: $II^+ = (3.75, 5.25)$ and $III^+ = (5.25, 3.75)$. The NAM region is virtually unaffected. The final PAM region begins at $\Omega > 0.49$, slightly larger than in Table S3.
