# Supplementary material for: An economic model and evidence of the evolution of human intelligence in the Middle Pleistocene: Climate change and assortative mating
Source: PLoS One. 2023 Aug 2;18(8):e0287964. doi: 10.1371/journal.pone.0287964 (PMC10395973; doi:10.1371/journal.pone.0287964)
Supplement: S4 File — (PDF) [file pone.0287964.s005.pdf]

S4: Mating patterns for alternative trait values

Table S4 provides a sense of how mating patterns vary as trait values change. The trait values given below (i.e., $2 \leq T \leq 5$), along with the previous examples, cover the range of reasonable values given the manner in which complementarities are modeled (i.e., the multiplication of T values). The first column of the table gives the trait values for the two key types: I and III. As in Tables S2 and S3, $\rho = 0.9$.

Table S4: ***Mating Patterns for Alternative Trait Values (ρ = 0.9)***

Traits (S, T) for: I and III	Initial PAM	NAM	Final PAM region
(2, 4); (4, 2)	$\Omega < 0.20$	$0.20 < \Omega < 0.61$	$\Omega > 0.61$
(2.5, 3.5); (3.5, 2.5)	$\Omega < 0.24$	$0.24 < \Omega < 0.53$	$\Omega > 0.53$
(2.5, 5); (5, 2.5)	$\Omega < 0.22$	$0.22 < \Omega < 0.54$	$\Omega > 0.54$
(3, 4.5); (4.5, 3)	$\Omega < 0.27$	$0.27 < \Omega < 0.44$	$\Omega > 0.44$

In the first row, Type I has traits of (2, 4) and Type III has traits of (4, 2). Compared to the example reported in Table S3 in the paper (e.g., Type I = (3, 6)), lowering the absolute size of trait values dampens the gain from complementarities, with the greatest effect on the Type I pairing. Thus, the NAM region expands to $0.20 < \Omega < 0.61$, somewhat larger than in Table S3 when $\rho = 0.9$. In the next row, the gap in traits between Type I and III is shrunk by 50%, reducing the gains from specialization. This shrinks the NAM range, to $0.24 < \Omega < 0.53$, which is still substantial in size. The final two rows begin with Type I = (2.5, 5) and Type III = (5, 2.5) and then again shrinks the gap in traits. Even at (3.0, 4.5) and (4.5, 3.0), there is still a considerable NAM region. Thus, within the confines of the model, for not particularly large trait differentials between Types I and III, along with moderate complementarities for public goods production, NAM occurs for a substantial range of Ω.

The mating patterns do not depend on hours available for each mate. Hours do, however, impact *CHILD*. If, for example, Type I is set at (2, 4) and Type III at (4, 2), hours set at around $H^f = H^m = 2$ generates roughly similar *CHILD* production as the numbers in Table S2B.

Finally, it is useful to consider what happens for outliers to Type I. Depending on the genetic lottery, offspring from a Type I pairing can be somewhat more intelligent than Type I (e.g., Type I^+). Importantly, in the model, during a sufficiently adverse climate, Type I^+ have higher utility and reproductive fitness if they pair with another Type I^+. Thus, in a sufficiently adverse climate, the evolution of intelligence is favored not only because the Type I pairing has the highest fitness, but positive outliers should select other positive outliers. Returning to the example in S2, let Type I^+ to be (2.7, 6.3), or 5% more intelligent and 10% weaker than Type I. At a severe climate of $\Omega = 0.7$, a Type I^+ pairing will have approximately 3% higher *CHILD* than the Type I pairing.
