# Supplementary material for: An economic model and evidence of the evolution of human intelligence in the Middle Pleistocene: Climate change and assortative mating
Source: PLoS One. 2023 Aug 2;18(8):e0287964. doi: 10.1371/journal.pone.0287964 (PMC10395973; doi:10.1371/journal.pone.0287964)
Supplement: S5 File — (PDF) [file pone.0287964.s006.pdf]

S5: Only public goods' exponents vary with climate shocks

In the examples in the paper, the utility and production functions are assumed to exhibit constant returns to scale. How sensitive are the results if climate change impacts only the exponents for public goods? To get a feel for this, fix the exponent for private goods in the *CHILD* production function at $\gamma = 0.65$, and the exponent for private goods in the utility function at $\beta = \gamma/2 = 0.325$ (since $\theta = 0.5$). This is a reasonable choice as these are exactly the values for the intermediate climate in Table S1. Thus, as climate varies (given by K_C and K_A), only the exponents for public goods in the model vary, where $\Omega_k = 1 - K_C$ and $\alpha_k = \Omega_k/2$. (For example, when $K_C = 0.5$, $\Omega_k = 1 - K_C = 0.5$ and $\alpha_k = 0.25$, together with $\gamma = 0.65$ and $\beta = 0.325$.) So, the relative importance of public goods compared to private goods still rises as the climate deteriorates, just not as quickly as when the exponents in the *CHILD* production function and the utility function sum to one. Compared to Table S3, the size of the NAM region should increase because, with the exponents on private goods fixed, the value of private goods is somewhat lower in a benign climate and somewhat higher in an adverse climate. This increases the range over which specialization by I and III dominates PAM. As expected, the size of the NAM region increases compared to the ranges in Table S3. For example, using the traits used for Tables S2 and S3 above, when $\rho = 0.90$, the NAM region is $0.20 < \Omega < 0.61$, compared to $0.24 < \Omega < 0.49$ in Table S3. Furthermore, one could also fix the coefficient for α_k, and only allow Ω_k to increase: the NAM region becomes modestly larger than in the example noted above.
