# Supplementary material for: An economic model and evidence of the evolution of human intelligence in the Middle Pleistocene: Climate change and assortative mating
Source: PLoS One. 2023 Aug 2;18(8):e0287964. doi: 10.1371/journal.pone.0287964 (PMC10395973; doi:10.1371/journal.pone.0287964)
Supplement: S6 File — (PDF) [file pone.0287964.s007.pdf]

S6: Allowing intelligence to matter for private goods production

The basic model can be extended to allow for intelligence to also impact private goods production. Consider the following production function for private goods:

$$PRIV = K_P * \left[h_{priv}^{m} * S_m * {T_m}^{\psi_k} + h_{priv}^{f} * S_f * {T_f}^{\psi_k}\right]$$

Compared to the private-goods production function in equation (4) in the paper, the main difference is that now intelligence is interacted with strength. Intuitively, if intelligence matters, it should matter more as the climate becomes more challenging; that is, ψ_k increases as the climate deteriorates. There is also a climate shock K_P, where K_P declines as the climate deteriorates (keeping private goods production from necessarily rising with deteriorating climate).

Compared to the main model, the value of Type I as a mating partner rises faster as the climate deteriorates given that now intelligence also matters for private goods production and ψ_k increases as the climate deteriorates. This means the NAM region should shrink. To get a sense of how much, set $K_P = K_A = K_C$ and $\psi = 1 - K_P$. That is, K_P takes on the same value as the other climate shocks and let ψ_k rise lockstep with the deterioration in climate, taking on the same values as Ω_k. Selecting the same trait values as used in Table S3, when $\rho = 0.90$, the NAM region is $0.22 < \Omega < 0.34$, a substantial shrinkage. There is also a transitional region where it is optimal for I and II to pair bond ($0.34 < \Omega < 0.38$). PAM then begins at $\Omega > 0.38$.
