# Supplementary material for: An economic model and evidence of the evolution of human intelligence in the Middle Pleistocene: Climate change and assortative mating
Source: PLoS One. 2023 Aug 2;18(8):e0287964. doi: 10.1371/journal.pone.0287964 (PMC10395973; doi:10.1371/journal.pone.0287964)
Supplement: S7 File — (PDF) [file pone.0287964.s008.pdf]

S7: Defense of the use of utility functions (reproductive fitness)

A key element of the model is a utility function with arguments beyond children. Evolutionary biologists (see Ridley [110] for a review) have historically assumed that human behavior is hardwired by natural selection to maximize reproductive fitness. However, as reviewed by Sear et al. [128], many studies now challenge this view, noting the dramatic declines in global birth rates and the fact that total fertility does not appear to be fitness-maximizing even in natural-fertility societies. Economists take an approach that is different from evolutionary biologists (see Robson and Samuelson [129] for an overview).

Based on the tremendous climate swings (see Fig 2 in the main text) over the last 700 kyr, the importance of family public goods in the *CHILD* production function (captured by Ω_k) likely changed repeatedly and dramatically. In the chaotic climate of the Middle Pleistocene, genetically programmed rules arguably would perform very poorly. In particular, upon entering a severe glacial phase, they would initially sharply under produce family public goods, harming reproductive fitness. It seems plausible that Middle Pleistocene humans, of even modest intelligence, could make better decisions based on utility functions. For example, consider a pair-bonded couple that suffered through several unusually frigid nights. They would recognize that their young children are in great danger, providing strong impetus for an improved shelter. In the model in the paper, parents have children in their utility function, which amounts to having the productivity of family public goods for producing surviving children in the utility functions (see equation 3 in the main text).

To obtain additional insight, consider how production decisions characterized by equations 7.1 and 7.2 in the main text differ from the production "decisions" of parents genetically hardwired to maximize reproductive fitness. (Hardwiring is the correct notion because why else would hominins ignore benefits from consuming other goods?) This is found by maximizing utility where the only argument in the utility function (equation 2 in the main text) is *CHILD* and where $CHILD = K_C * PUB^{\Omega} * PRIV^{\gamma}$. The optimal mix of public and private goods is:

$$PUB/PRIV = \Omega_k * (T_m * T_f)^{\rho} / \left(\gamma_k * (S_m + S_f)\right).$$

Compared to equation (7.2) in the main text, the main difference is that now all private goods are allocated to children (which of course is not possible in practice).

Suppose that for thousands of years, the climate is benign as given by K_C = 0.85 and $\Omega_k = 1 - K_C = 0.15$. Using the equation above and making the same assumptions concerning traits, available hours, and complementarities used for computing the numbers in Tables S2 and S3 above, the number of children produced by the Type I pairing is exactly 4.14. (Since zero private goods are allocated to parents, this number is artificially inflated compared to the number of children in the first cell of Table S2B.) Suppose suddenly the climate changes to $K_C = 0.45$ and $\Omega_k = 0.55$, the adverse climate in Tables S2A, S2B and S3. Since a genetically hardwired rule is inflexible, assume that initially there is no increase in public goods. So, compute $CHILD = .45 * PUB^{.55} * PRIV^{.45}$ where *PUB* and *PRIV* are set at their optimal values when $\Omega_k = 1 - K_C = 0.15$. The result is that *CHILD* production for the Type I pairing falls from 4.14 to 1.94, a decline of 53%, far larger than if private and public goods could optimally adjust to the new climate. In contrast, in Table 2SB for the Type I pairing, switching from the benign to the adverse climate causes *CHILD* to fall from 2.30 to 2.18, only a 5.2% decline. Thus, even though utility maximization (with arguments beyond *CHILD*) does not maximize reproductive fitness, its flexibility in the face of climate fluctuations could cause it to ultimately have higher fitness than a genetically hardwired rule. This example supports Robson's [28] argument for why nature equipped individuals with utility functions, providing a defense for the use of utility functions in the paper.
