# Supplementary material for: An economic model and evidence of the evolution of human intelligence in the Middle Pleistocene: Climate change and assortative mating
Source: PLoS One. 2023 Aug 2;18(8):e0287964. doi: 10.1371/journal.pone.0287964 (PMC10395973; doi:10.1371/journal.pone.0287964)
Supplement: S8 File — (PDF) [file pone.0287964.s009.pdf]

S8: Crania Used in Fig. 3

The data used for Figure 3 appears in Table S5 below. I begin with the database compiled in De Miguel and Henneberg [104]. As commonly done, I report the average cranial size when there are multiple size measures and exclude juveniles. In addition, I searched for all crania new to the literature (with data on size and age) and added 13 to the sample. This includes five additional adult crania from the archeological site at Atapuerca (Sima de los Huesos), as well as the two *H. sapiens* crania from Morocco. After 700 ka, there are no *H. erectus* crania from Africa; there are, however, some *H. erectus* crania outside Africa (China and Indonesia). These are less relevant for the predictions, and they are not included in Table S5 (or in Fig. 3). Finally, I checked all crania for any modern re-dating. Precise hominin classification in the Middle Pleistocene is challenging. After 700 ka, the set of crania in Table S5 were referred to as "archaic humans" in De Miguel and Henneberg [104]; to reduce confusion, the crania after 700 ka (excluding *H. sapiens*), are labeled as *H. heidelbergensis*. Notice that there are *H. heidelbergensis* crania dating slightly after the two *H. sapiens* crania. This is not surprising, as a speciation event need not immediately lead to the extinction of earlier species, especially given the enormous geographic region occupied by *H. heidelbergensis*.

Table S5

Crania Used in Fig. 3				
ID	Taxon	Date	CC (ml)	Source
Modjokerto	erectus	1800	855	Miguel & Henneberg [104]
ER3733	erectus	1780	825.4	"
Dmanisi Skull 1	erectus	1770	730	Rightmire et al. [107]
Dmanisi Skull 2	erectus	1770	655	"
Dmanisi Skull 3	erectus	1770	600	"
Dmanisi Skull 4	erectus	1770	625	"
Dmanisi Skull 5	erectus	1770	546	"
Sangiran 31	erectus	1660	1000	Miguel & Henneberg [104]
KNM-WT	erectus	1600	904.5	"
ER3883	erectus	1570	825.7	"
OH9	erectus	1200	1070.5	"
Sangiran 9	erectus	1200	856	"
Gongwangling 1	erectus	1150	779	"
Sangiran 4	erectus	1000	856	"
Daka	erectus	1000	995	Asfew et al. [108]
Trinil 2	erectus	900	918.7	Miguel & Henneberg [104]
Sangiran 2	erectus	900	792.5	"

Sangiran 3	erectus	900	900	“
Sangiran 12	erectus	900	951	“
Sangiran 17	erectus	900	1020	“
Sangiran 10	erectus	850	868.6	“
OH 12	erectus	840	732.3	“
Bodo	heidelbergensis	600	1275	“
Ceprano	heidelbergensis	450	1185	Miguel & Henneberg [104]; Ayala and Cela-Conde [109] dating
Atapuerca 2	heidelbergensis	430	1333.5	Arsuaga et al. [12]
Atapuerca 4	heidelbergensis	430	1390	“
Atapuerca 5	heidelbergensis	430	1092	“
Atapuerca 12	heidelbergensis	430	1227.5	“
Atapuerca 13	heidelbergensis	430	1436.5	“
Atapuerca 15	heidelbergensis	430	1283.5	“
Atapuerca 17	heidelbergensis	430	1218.5	“
Arago 21	heidelbergensis	400	1138.7	Miguel & Henneberg [104]
Broken Hill 1	heidelbergensis	350	1310	“
Ndutu 1	heidelbergensis	350	1100	“
Sahlanha 1	heidelbergensis	350	1216.7	“
Petralona 1	heidelbergensis	300	1266.6	“
Steinheim 1	heidelbergensis	300	1111.2	“
Swanscombe 1	heidelbergensis	300	1305	“
Narmada 1	heidelbergensis	300	1249.3	“
KNM-ER3884	heidelbergensis	270	1400	“
Jebel Irhound 1	sapien	315	1375	Neubauer et al. [15]
Jebel Irhound 2	sapien	315	1467	“
