# Supplementary material for: An economic model and evidence of the evolution of human intelligence in the Middle Pleistocene: Climate change and assortative mating
Source: PLoS One. 2023 Aug 2;18(8):e0287964. doi: 10.1371/journal.pone.0287964 (PMC10395973; doi:10.1371/journal.pone.0287964)
Supplement: S9 File — (PDF) [file pone.0287964.s010.pdf]

S9: Additional References

121. de Boer B. Loss of air sacs improved hominin speech abilities. Journal of Human Evolution 2012; 62: 1-6.
122. de Boer B, Thompson B, Ravignani A, Boeckx C. Evolutionary dynamics do not motivate a single-mutant theory of human language. Scientific Reports. 2020; 10: 1-9.
123. Alperson-Afil N, Goren-Inbar N. The Acheulian Site of Gesher Benot Ya'aqov, Volume II. Dordrecht: Springer; 2010.
124. Gowlett, J.A., R.W. Wrangham, 2013. Earliest fire in Africa: Towards the convergence of archaeological evidence and the cooking hypothesis. Azania: Archaeological Research in Africa 48, 5-30.
125. Agam A, Azuri I, Pinkas I, Gopher A, Natalio F. Estimating temperatures of heated Lower Palaeolithic flint artefacts. Nature Human Behavior. 2020; 221-228.
126. Flinn MV, Geary DC, Ward CV. Ecological dominance, social competition, and coalition arms races: Why humans evolved extraordinary intelligence. Evolution and Human Behavior. 2005; 26: 10-46.
127. Jarvenpa, R., H.J. Brumbach, 2014. Hunter-gatherer gender and identity. In: Cummings, V. Jordan, P., Zvelebil M. (Eds.), The Oxford Handbook of the Archaeology and Anthropology of Hunter-Gatherers, Oxford University Press, Oxford, UK., 1243-1265.
128. Sear R, Lawson DW, Kaplan H, Shenk MK. Understanding variation in human fertility: What can we learn from evolutionary demography? Philosophical Transactions of the Royal Society B Biological Sciences. 2016; 371: 1-15.
129. Robson AJ, Samuelson L. The evolutionary foundations of preferences. In: Benhabib J, Bisin A, Jackson M, editors. Handbook of social economics. Amsterdam: North-Holland; 2011. pp. 221-310.
